# Artificial intelligent fuzzy control and LAPO algorithm for enhancement LVRT and power quality of grid connected PV/wind hybrid systems

**DOI:** 10.1038/s41598-024-78384-5

**Published:** 2024-12-16

**Authors:** Noura G. EL Sayed, Ali M. Yousef, Gaber El-Saady, Meshari D. Alanazi, Hamdy A. Ziedan, Montaser Abdelsattar

**Affiliations:** 1 Department of Electrical Engineering, Faculty of Engineering, Assiut University, Assiut, 71516 Egypt; 2 Department of Electrical Engineering, College of Engineering, Jouf University, Sakaka, 72388 Saudi Arabia; 3 Electrical Engineering Department, Faculty of Engineering, South Valley University, Qena, 83523 Egypt

**Keywords:** Low voltage ride through (LVRT), Static synchronous compensators (STATCOM), Wind, Photovoltaic (PV), Proportional–integral–derivative (PID) controller, Proportional–integral fuzzy logic control (PI FLC), Lightning attachment procedure optimization algorithm (LAPO), Power quality, Engineering, Electrical and electronic engineering

## Abstract

Low Voltage Ride Through (LVRT) is considered one of the main and serious problems facing the electrical grid. It occurs due to three-phase symmetric faults and asymmetric faults such as a double line to ground fault that applies in this system. This paper applies Static Synchronous Compensators (STATCOM) to improve the LVRT capability and dynamic performance of an electrical grid linked to a Photovoltaic (PV)/Wind hybrid system through grid disturbances. A hybrid power system containing a PV station that produces 1 MW and a wind farm from type Doubly Fed Induction Generator (DFIG) that produces 9 MW is connected to STATCOM with 48 pulses at PCC bus and energized load. It compensates reactive power to improve LVRT that occurred due to fault. The applied STATCOM controller adjusts the voltage of the PCC bus during an occuring fault on the grid by compensating reactive power. STATCOM is controlled by a Proportional–Integral–Derivative (PID) and is compared with STATCOM controlled by Artificial Intelligence Control (AIC)-based on Proportional—Integral Fuzzy Logic Control (PI FLC). The Lightning Attachment Procedure Optimization Algorithm (LAPO) optimization method is used to adjust the parameters of the PI controller to reduce error signals. A simulation model of the suggested hybrid power system has been performed using Matlab/Simulink. The simulation results of STATCOM proved powerful and the effectiveness of STATCOM with PI FLC in reducing voltage dip, compensating active power of wind and PV farm, protecting DC-link voltage of PV and wind from overvoltage and oscillation that happens at three-phase fault and double line to ground fault as compared with PID STATCOM in enhancement LVRT capability, and power quality.

## Introduction

The increasing demand for sources of energy, renewable energy resources were being used in place of fossil fuels. Fossil fuel sources are very costly, and stocks are finished^1^. Sources of renewable energy such as wind, PV, and biomass energies^2^.

Low Voltage Ride Through (LVRT) improvement is very important to protect the system from voltage dip^1^. Solar and winds are the fastest rising in the production of power sources. Although successful generation of renewable energy sources is still facing problems. The deviation in solar irradiation levels causes enormous fluctuations in power generation^3–9^.

In Ref.^4^ control construction of hybrid system PV and wind connected to grid was designed. The maximum power point tracking method was utilized to obtain maximum power from the subsystem. The control method regulated DC-link voltage, maintained the voltage of the grid constant, and controlled power fed into the grid. Also, a phase-locked loop was used for the synchronization of the grid.

In Ref.^5^ the control method and optimization method were introduced a hybrid Firefly Algorithm-Particle Swarm Optimization (FA-PSO) was utilized to detect. Fractional Order Proportional Integral-Derivative (FO-PID) control, Takagi Sugeno Fuzzy Inference Systems (TSFIS), and Adaptive Neuro-Fuzzy Inference Systems (ANFIS). The results cleared the superiority of the (FA-PSO) optimized ANFIS-PID controller method compared to fuzzy PI and PID controller. This superiority was clear in DC-link voltage transient response through system disturbances, achieving faster settling time and less deviation.

In Ref.^6^ the deloading based on active power control of variable speed DFIG wind turbines was proposed for regulating primary frequency. To deal with a nonlinear curve of power characteristics for wind turbine generators, a Lagrange interpolating polynomial was presented. Utilizing the suggested Lagrange interpolating polynomial based on deloading, the overall capacity factor of the wind farm was enhanced, and the fuel consumption of the diesel generator decreased for the same power demand.

In Ref.^7^ a Unified Power Quality Conditioner (UPQC) was proposed under two conditions the first condition of nonlinear load and the second unbalanced load. It was used to improve the power quality of the system. It is composed of two voltage source inverters placed in series and a shunt in the system. The classic control PI was utilized to mitigate voltage sagging, swelling, and harmonic. Also, an intelligent control method based on a TS-fuzzy algorithm was introduced. The results of the simulation cleared that the method of TS-fuzzy effective in reducing voltage sagging, swelling, and harmonic distortion compared with PI controller in improving power quality.

In Ref.^8^ The LVRT problem occurs when it is near the fault of the grid. In this case, it causes a reducing in the voltage of the grid when the point of the generator is connected to the grid this leads to limited power that can be generated from the device.

In Ref.^10^ many devices were being used to enhance LVRT capabilities, such as braking resistors, DC chopper, series dynamic braking resistors, fault current limiters series, and crowbar circuits. These methods involve decreasing DC-link voltage, voltage dips, torque fluctuations, and over-current.

In Refs.^11–13^ also other devices were suggested to solve the problem of LVRT, in these methods voltage of the grid was regulated by using devices such as Static Synchronous Compensators (STATCOM), Synchronous Series Compensators (SSSC), Dynamic Voltage Restorers (DVR) and Thyristor-Controlled Series Compensators (TCSC). These methods involve adjusting active and reactive power to enhance LVRT.

Ref.^14^ was proposed comprehensive impedance modeling of the Induction Motors (IMs), Machine Side Converter (MSC), Grid Side Converter (GSC), and grid to dissect the stability of the system although Constant Power Loads (CPL) operation and transmission line failure mode operation. The oscillations were reduced by detecting Phase-Locked Loop (PLL) bandwidth or by producing the Battery Energy Storage System with a Static VAR Compensator (BESS-SVC) unit into the load bus.

In Ref.^2^ Flexible AC Transmission System (FACTS) plays a serious role in voltage sag compensation and voltage stability enhancement. STATCOM is one of the most important methods for improving transient stability by compensation of reactive power at nominal bus^3^. Controllers STATCOM-based Artificial Intelligence (AI) such as Artificial Neural Network (ANN) and Fuzzy Logic Control (FLC) based controller were presented in Refs.^4–16^.

LVRT capability is one of the most important grid code requirements, which means that renewable energy systems must stay linked through faults that occur in the grid and provide an electrical grid for reactive power^17^. The possibility of power production from hybrid systems is confirmed to be very favorable and credible^18^. Ref.^19^ presented STATCOM joined to a small series dynamic braking resistor to improve grid stability linked to a wind farm consisted of a fixed-speed wind turbine generator system. FLC has shown great performance in nonlinear system tuning^18^. Some studies have examined the means of raising the output of power and enhancing PV systems for providing active power to the power grid, but few have shown power quality problems such as harmonic reduction, voltage sagging, phase unbalance, over-voltage, under-voltage and power factor deterioration. Power converters are the primary means of converting power from PV systems to power from the grid^18^. In Ref.^19^ the multi-objective LAPO calculated the best parameter of PI STATCOM controller. The major goal was improving combined wind farm performance which was based on a consisting of DFIG and SCIG using the STATCOM tuned by LAPO^19,20^. Combined wind farm performance with STATCOM tuned by LAPO has better performance than DFIG and SCIG wind farm with STATCOM tuned by LAPO through asymmetric line-to-line and symmetric three-phase fault^19,20^.

Control designing of LVRT capability for single-stage inverter-based network linked to PV system was introduced. Control designing was presented to reduce problems of DC-link over-voltage and AC over current that causes deterioration of inverter when fault occur on grid^21^. Crowbar is one of the most widely used methods for enchantment Fault-Ride-Through (FRT) capability^19^. The chopper method and capacitors were used to regulate the voltage of the DC connection^21,22^. Dynamic brake resistors and fault-current limiters^23,24^ are two other methods proposed for reducing over current of the rotor and stator. DFIG-based wind turbines are very critical to disturbances of grid voltage. Voltage dip and active power production decrease when a fault occurs that leads to fast rise in a current of the rotor to compensate for the active power^21–23^.

The Dynamic Voltage Restorer (DVR) was introduced to reduce stator voltage when a fault of the grid occurs^24,25^. FACTS devices were used to improve DFIG and PV LVRT capabilities^26^. Through grid disturbances, these systems supply additional reactive power to the electrical grid to restoration of grid voltage^27^. In Ref.^10^ improving the crowbar configurations method by using Pulse Width Modulation (PWM) that is controlled by Insulated-gate bipolar transistor (IGBT) bridges leads to improving the performance of LVRT by converting high rotor currents away from power electronics. Series Grid-Side Converter (GSC) control strategy was developed to display active and reactive power support through LVRT, thereby reducing oscillations of DC-link voltage, oscillations of stator voltage, and high stator currents. In Refs.^11–28^ the suggested approach include using of flying capacitors storage capacity to store excess energy produced through grid faults. Controller design objectives include improving energy extraction from the turbine, adjusting DC-link voltage, preserving exact control of flying capacitor voltages, and mitigation voltage dips through a symmetrical fault to enhance LVRT^28,29^.

In Ref.^30^ the proposed method is optimizing tuning of the STATCOM controller by using the optimization method of hybrid ACO-PSO, that method was used to adjust a parameter of the PI controller of STATCOM. Also, the STATCOM PI controller is optimized by the Sine-Cosine Algorithm (SCA) to enhance power system stability.

In Ref.^31^ LVRT capability of DFIG-based wind farms was enhanced by an optimized PI STATCOM controller and FOPI-PI STATCOM controller. In Ref.^32^ the enhancement of LVRT capability by enhancing the grid-side inverter performance and also reinforcement of DC-DC boost converter action while preserving an enhanced range of steady-state error, settling time, and overshoot. In Ref.^33^ the proposed method of a cascaded adaptive fuzzy logic control was used to detect the grid-side converters and rotor-side to improve the performance of the based Wind energy conversion system. The suggested methods can detect active and reactive power in the network through disturbances to improve LVRT.

In Ref.^34^ the proposed method of external resistance and strategy of demagnetization control in the DFIG rotor and stator side is to improve the LVRT capability of the DFIG-based wind energy conversion system. As well on the stator side, the external resistance accelerated the damping of transient flux by reducing constant time. For demagnetizing control, the transient response of electromagnetic torque, DC-link voltage, stator, and rotor current, of DFIG-based wind energy conversion systems are clearly improved at initiation and redemption at grid faults.

In Ref.^35^ the proposed method for controlling LVRT-based virtual reactance voltage control. This controlling method regulated voltage, active, reactive power, and current during faults in a grid. The experimental results and simulation proved the effectiveness of the proposed method in enhanced LVRT.

In Ref.^36^ the proposed method of LVRT control is based on fault I-V characteristics of Virtual Synchronous Generator (VSG). Transient stability analysis was performed to ensure synchronization between the power grid and VSG, especially when critical grid disturbances occur. The reactive power was raised with the introduced I-V characteristics of the Virtual Synchronous Generator (IV-VSG) control to support power grid voltage. The active power was reduced to reduce fault current improve transient stability and enhance the control method to increase the LVRT capability of Grid-Tied Converters under different grid-fault.

In Ref.^37^ the proposed demagnetization control method in DFIG and stator dynamic model were developed to provide LVRT capability. The stator dynamic model was designed to reduce disturbances that happen due to the stator being connected directly to the machine and increase the performance of calculation in the machine. However, forced flux and natural models based rotor electromotive force that developed in demagnetization control method in DFIG. Method of demagnetization control developed for symmetrical and asymmetrical faults that give better results than the traditional model in terms of stability and oscillations.

In Ref.^38^ the Mountain Gazelle Optimizer (MGO) was proposed to adjust the proportional-integral (PI) controller parameters of the DFIG system reactive and active power control to improve the LVRT capability of wind turbines that connected to a power grid. In the introduced sketch, LVRT enhancement was proportional to settlement time, overshoot, undershoot, and steady-state inaccuracy of voltage responses. The proposed MGO method’s effectiveness was cleared by comparison to classic optimization based on PI controllers under different faults. The result cleared that the method of optimizing control enhanced the performance of DC link voltage, three–phase terminal voltage, and active and reactive power.

In Ref.^39^ proposed methods are the stator–rotor electromotive force (emf) model was developed in the RSC circuit of DFIG, a lookup-table-based supercapacitor model was developed in the GSC circuit of DFIG, and STATCOM model connected to a grid of DFIG-based on a wind turbine was utilized to the analysis of three phases and two-phase fault. Developing the stator model leads to increasing the performance of the simulation study. While the rotor model leads to reducing oscillations of the system and the time to reach the system stability. Developing supercapacitor model based on the lookup table, both appropriate capacity and smooth power output for the appropriate voltage were obtained. Also decoupled STATCOM compensated bus voltages that depend on the reactive power in transient stability analysis. In Ref.^40^ STATCOM was proposed to improve the performance of hybrid PV and wind systems during three-phase faults. STATCOM is based on two PI controllers to regulate reactive power and regulate the voltage at PCC bus. In Ref.^41^ introduced the Taguchi method to determine optimal allocation (size and location) and control parameters of STATCOM to improve the LVRT capability of wind farms. The control gains of STATCOM are tuned using fuzzy control. Analysis of Mean (ANOM) technique that part of the Taguchi method determined fuzzy control parameter of STATCOM and allocation to obtain robust design that is insensitive to variations of fault and operating conditions. However, the STATCOM device has many advantages in power systems, especially in improving power quality, voltage stability, and LVRT^41^. There are many advantages like voltage regulation where STATCOM can detect and control the voltage, mitigate voltage sagging, swelling, fluctuations, and flickering to improve power quality, respond quickly to sudden changes and faults to decrease oscillations and achieve grid stability, STATCOM has low losses compared to other systems like static VAR Compensator (SVC), improving the overall efficiency of the system, Also STATCOM has long service life and low maintenance.

This paper studies the problem of LVRT and its effect on the system. So 100 MVAR STATCOM with 48 pulses is used to overcome this problem. STATCOM is controlled by PID and is compared with STATCOM controlled by Artificial Intelligence Control (AIC)-based on PI FLC. The LAPO optimization method is used to adjust the parameters of the PI controller to reduce the error signal. The simulation results of STATCOM proved powerful and the effectiveness of STATCOM with PI FLC in reducing voltage dip, compensating active power of wind and PV farm, protecting DC-link voltage of PV and wind from overvoltage and oscillation that happens at three-phase fault and double line to ground fault as compared with PID STATCOM in enhancement LVRT capability, and power quality. So this method gives the best results compared to the previous studies. This paper introduces a motivation for improving LVRT in PV/wind hybrid systems through grid disturbances. The hybrid system consists of a 1 MW PV station and 9 MW wind farm from type DFIG which is linked to STATCOM that provides for 100 MVAR at PCC bus to compensate reactive power. STATCOM controller-based PID control and PI FLC to improve LVRT. Also, the LAPO optimization method is used to adjust a parameter of the PI controller to reduce the error signal. LVRT occurs due to three-phase symmetric faults and asymmetric faults such as a double line to ground fault that applies in this system. Also, this paper applies STATCOM to improve LVRT and power quality. Finally, the main contributions of this paper are listed as follows:

- Discussing the problem of LVRT and its effect on the electrical grid connected to PV/wind hybrid system.
- Applying 100 MVAR STATCOM linked to a hybrid system that consists of a 1 MW PV station and 9 MW wind farm to improve LVRT, voltage stability, and power quality.
- STATCOM controller based on PID control and PI FLC to improve LVRT capability and dynamic performance of electrical grid linked to PV/Wind hybrid system.
- The LAPO optimization method is used to adjust the parameters of the PI controller to reduce the error signal.
- Comparison between the PID STATCOM controller and PI FLC STATCOM controller confirms the effective performance of the STATCOM controller in the enhancement of LVRT capability.
- Results of the simulation show that the applied STATCOM reduces voltage dip, compensate for active and reactive power of wind and PV farm, and protect the DC-link voltage of PV and wind from overvoltage and oscillation.

This paper is arranged as follows; “Suggested hybrid system PV/wind with STATCOM” section introduces the suggested hybrid system PV/Wind with STATCOM. “Fuzzy PI control” section presents fuzzy PI control. LAPO optimization” section presents LAPO optimization, “Control circuit of STATCOM” section introduces the control circuit of STATCOM with PID controller, “Simulation results and discussion” section presents simulation results and discussion, and “Conclusion” section presents the conclusion.

## Suggested hybrid system PV/wind with STATCOM

**Fig. 1:**
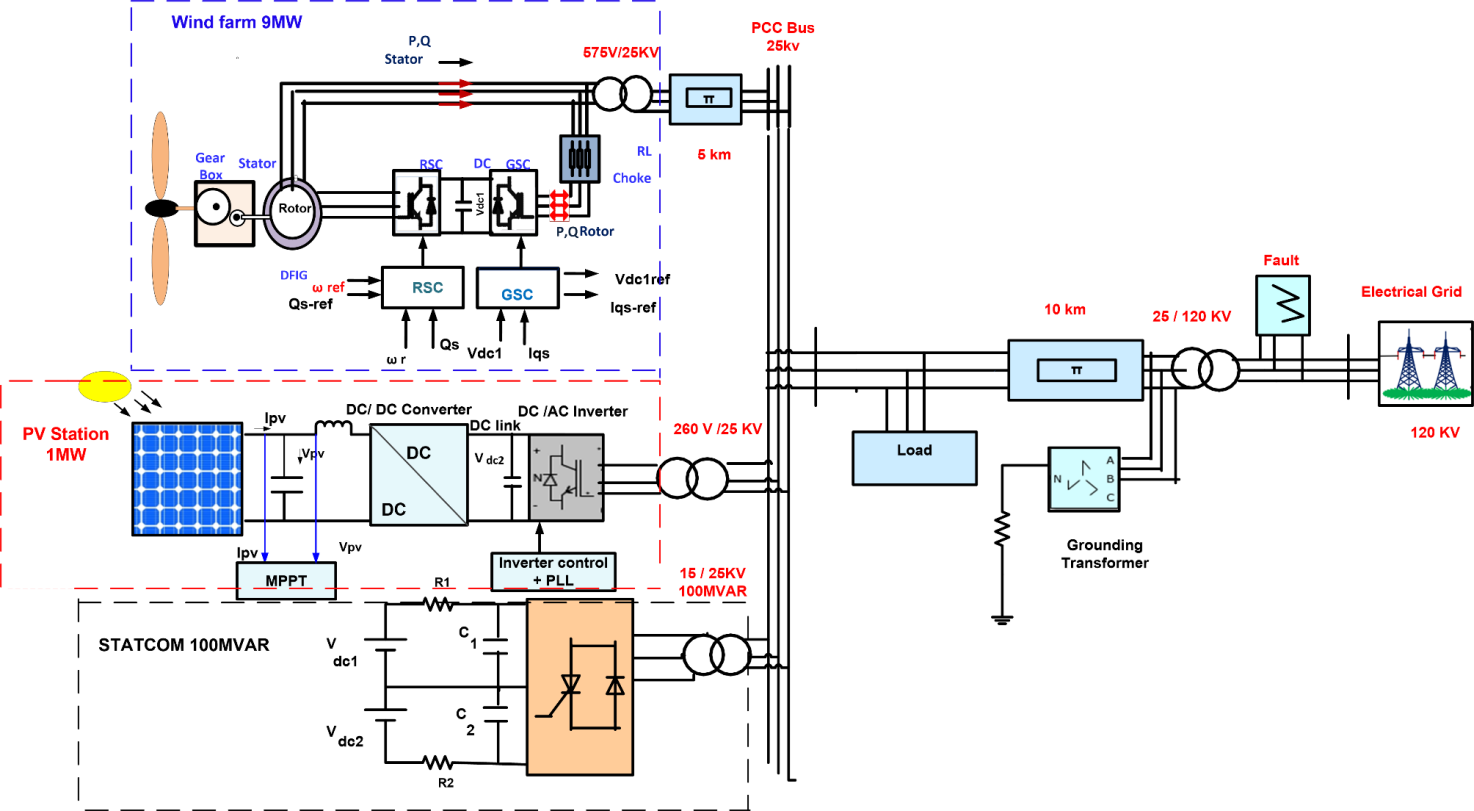
PV/Wind Hybrid renewable power system configuration with STATCOM.

The suggested hybrid system connected to the electrical grid consists of a 1 MW PV station and 9 MW wind farm from type DFIG which is linked to STATCOM that provides for 100 MVAR at PCC bus as shown in Fig. 1. Wind farm produced 9 MW contains six variable-speed wind turbines that produce 1.5 MW^21^. DFIG wind farm consists of RSC to catch maximum power through the variation speed of the wind and GSC to adjust the reactive power with the electrical network^25^, on the other hand PV station that produces 1 MW contains 640 parallel-linked to strings of PV, each PV string consisted of five PV panels linked in series^27^. Also as illustrated in Fig. 1, STATCOM is connected to a hybrid system during the bus of PCC to improve the LVRT capability and enhance voltage stability during occurring faults on a grid. The major aim of STATCOM is to adjust the bus voltage of PCC through voltage sagging by compensating reactive power. Parameters of system are shown in Tables 1, 2 and 3.

**Table 1:** Wind farm parameters.

Rated power of wind farm	9 MW	Magnatizing inductance (pu)	2.9
Rated power of wind turbine	1.5 MW	Fraction factor (pu)	0.01
Number of turbine	six	Number of pole pair	3
Generator type of turbine	DFIG	DC bus voltage regulator gains [Kp Ki]	[8 400]
DC bus voltage	1150 (V)	Grid-side converter current regulator gains [Kp Ki]:	[0.83 5]
Stator resistance	0.023	Rotor-side converter current regulator gains [Kp Ki]:	[0.6 8]
Stator leakage inductance (pu)	0. 18	Rated stator line-to-line voltage (V)	575
Rotor resistance	0.016	Cut-in wind speed	4
rotor leakage inductance (pu)	0.16	Rated wind speed	12
Reference voltage Vref (pu)	1	Cut-out speed (m/s)	20

**Table 2:** PV station parameters.

Rated power of PV Station	1 MW	Number of parallel-connected PV strings	640
Panel type	Sun power SPR-315E-WHT-D	Number of series-connected PV panels per string	5
Rated power of PV panel	315.072 W	Parameter of Voltage source converter (VSC) control of PV system	
Open circuit voltage	64.6 V	VDC regultator gains [ Kp Ki ]	[7 800]
Short circuit current of PV panel	6.14 A	Choke impedance [ R(ohm) L(H) ]	[0.1e-3 15e−6 ]
Standerd test condition	G = 1000 W/m^2^ ,T = 25, 45 ^o^C	Current regulator gains [ Kp Ki ]:	[0.2 20]
DC link voltage	500 V	Nominal primary and secondary voltages (V)	[ 25e3 260 ]

**Table 3:** STATCOM, electrical grid and load parameters.

Rated power of STATCOM	100 MVAR	Referance voltage pu	1
Number of zigzag phase-shifting transformer	4	Capacitance	3000 µF
Primary (zig-zag) nominal voltage Vp (VrmsPh-Ph):	25e3/4	Electrical Grid parameters	
Secondary nom. voltage phase shift	[ 15e3 +7.5]	Rated voltage	120 KV
Resistance of zig-zag winding [R L] (pu)	[0.05/30 ]	Three-Phase Series RL Branch	[2 43e−3]
Inductance of zig-zag winding L (pu)	0.05	Load parameter	
Magnetizing resistance (pu)	500	Active power of load	9.1 MW
Magnetizing inductance (pu)	500	Reactive power of load	2 MW

### PV station model

**Fig. 2:**
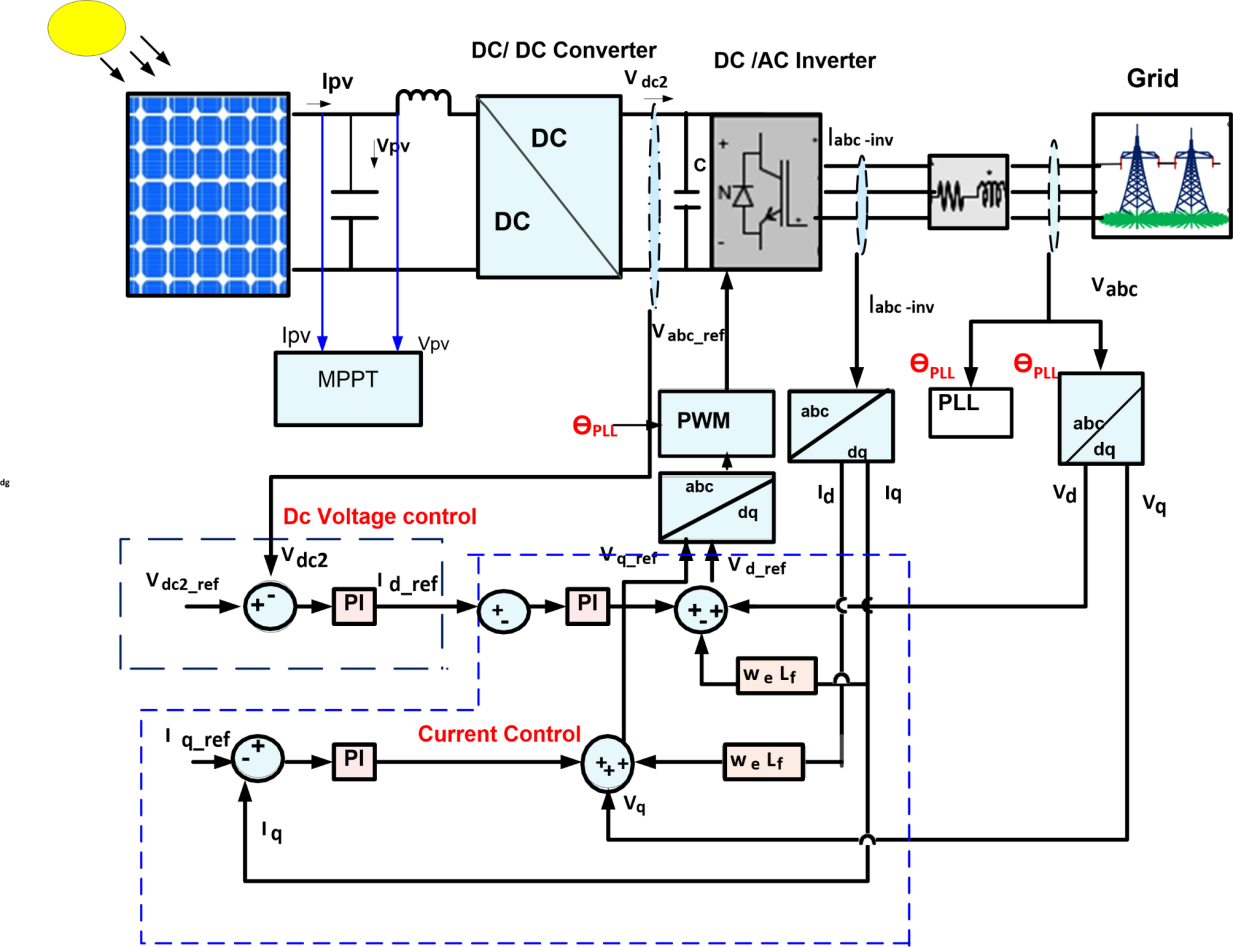
Configuration of the DC/AC inverter control for PV model.

**Fig. 3:**
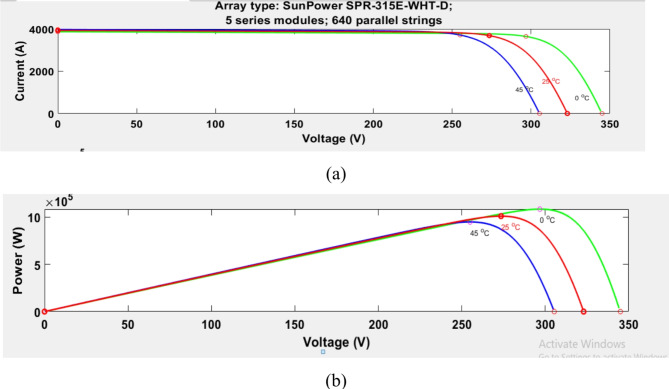
(I-V) and (P-V) curves characteristics of PV. (**a**) Characteristics (I-V) of PV. (**b**) (P-V) curves characteristics of PV.

PV is the best alternative choice for producing electricity from solar energy without emission of greenhouse gases, long lifespan, low maintenance, and good efficiency, among the abundant renewable sources^10,42,43^. Solar cells produce electrical power from the photon impact of sun irradiance^28^. The proposed PV station contains five PV modules linked in series to PV strings and a total of 640 PV strings that are linked in parallel to generate 1 MW. Sun power SPR-315E-WHT-D PV panel with a maximum power of 315.072 W is utilized for this work^43^. Figure 2 shows the control circuit of the PV model, and Fig. 3a and b shows the (I–V), and (P-V) characteristics^44^. The equation of the single diode of the PV system and inverter voltage equation in the d–q synchronous rotating reference frame is characterized as follows:

1 $$\:{I}_{pv}={I}_{ph}-{I}_{d}-{I}_{sh}={I}_{ph}-{I}_{o}\left[\text{exp}\left(\frac{{V}_{ph}+{I}_{pv\:}{R}_{s\:}}{\text{a}}\right)-1\right]-\:\frac{{V}_{ph}+{I}_{pv\:}{R}_{s\:}}{{R}_{sh}}$$

2 $$\:\text{a}=\frac{\text{n}\text{k}\text{T}}{\text{q}}$$

3 $$\:{V}_{d-inv}={V}_{d}+{{R}_{f}I}_{d}+{L}_{f}\frac{\text{d}{I}_{d}}{\text{d}\text{t}}-{\omega\:}_{e}{L}_{f}{I}_{q}$$

4 $$\:{V}_{q-inv}={V}_{q}+{{R}_{f}I}_{q}+{L}_{f}\frac{\text{d}{I}_{q}}{\text{d}\text{t}}-{\omega\:}_{e}{L}_{f}{I}_{d}$$

5 $$\:{\:\:\:\:\:(P}_{pv}-{\:\:\:\:\:P}_{g-f})\varDelta\:t={0.5{C}_{2}({V}^{2}}_{dc2-f}-{{V}^{2}}_{dc2})$$

6 $$\:{\:\:\:}_{Vdc2-f=x=\frac{\sqrt{2\left({P}_{pv}-3{V}_{f}{I}_{g}\right)\varDelta\:t}}{\text{C}2\:}+{{V}^{2}}_{dc2}}\:$$

### Wind modeling

**Fig. 4:**
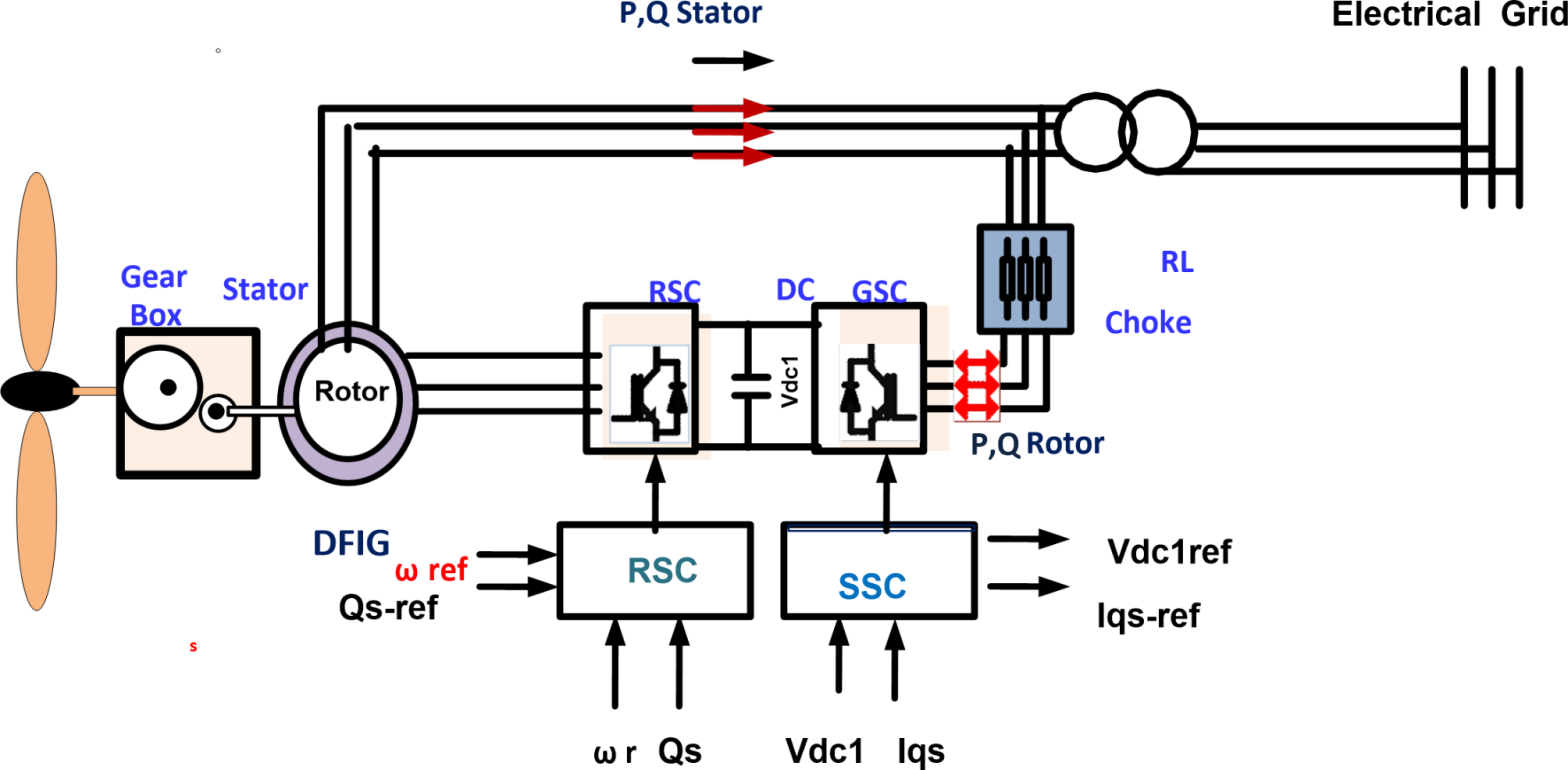
Configuration of wind system.

Figure 4 shows a model of a DFIG wind turbine that can be represented by the equations below. The stator winding is linked directly to the electrical network^12–45^. Each wind turbine was extended with the DFIG inclusive control of GSC to adjust reactive power with the electrical grid and control of RSC to catch the peak power during the wind speed deviation. A wind farm is linked with a bus of PCC during 0.575/25 KV Δ/Y step-up transformer to inject produced power into the electrical network^45,46^.

7 $$\:{P}_{m}=\frac{1}{2}{C}_{p}\left(\lambda\:,\beta\:\right){v}_{w}^{3}{\rho\:A}_{t}$$

8 $$\:{C}_{p}\left(\lambda\:,\beta\:\right)=\text{C}1\left(\frac{\text{C}2}{{\lambda\:}_{i}}-\text{C}3\beta\:-\text{C}4\right){\text{e}\text{x}\text{p}}^{\left(\frac{-\text{C}5}{{\lambda\:}_{i}}\right)}+C6\lambda\:\:\:\:$$

9 $$\:\frac{1}{\:{\lambda\:}_{i}}=\left(\frac{1}{\:\lambda\:+0.08\beta\:}\right)-\left(\frac{0.035}{\:{\beta\:}^{3}+1}\right)$$

10 $$\:{P}_{m}-\text{P}\text{e}=\frac{2{H}_{g}}{{\omega\:}_{s}}\frac{{d}_{\delta\:}^{2}}{{d}_{t}^{2}}$$

#### Grid side converter control

**Fig. 5:**
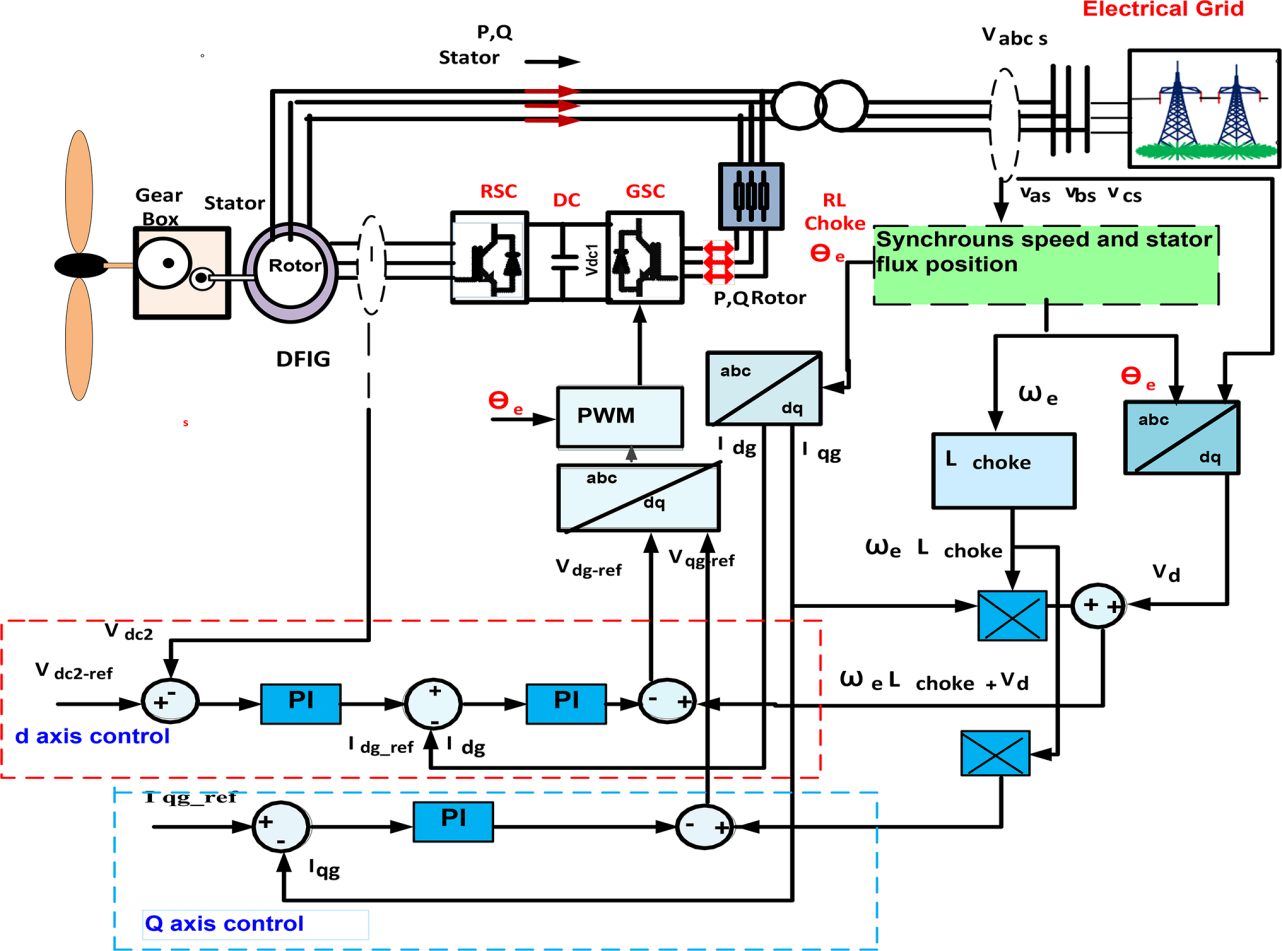
Control circuit of grid side converter of wind turbine.

Figure 5 shows control circuit of GSC was used to preserve voltage of DC bus, Vdc_2_ constant and adjust reactive power interchanged with electrical network. Also, reactive power injected from GSC,$\:\:{Q}_{g}$ was controlled with $\:{i}_{qg}$, while active power injected from GSC, $\:{P}_{g}$ and V_dc2_ was detected during $\:{i}_{dg}$, as expressed in the following Equations^46,47^.

11 $$\:{v}_{dg-ref}={-v*}_{dg}-{\omega\:}_{e}{L}_{choke}{i}_{qg}+{v}_{d}$$

12 $$\:{v}_{qg-ref}=-{v*}_{qg}-{\omega\:}_{e}{L}_{choke}{i}_{dg}$$

13 $$\:{P}_{g}=\frac{2}{3}({v}_{dg}{i}_{dg}+{v}_{qg}{i}_{qg})$$

14 $$\:{Q}_{g}=\frac{2}{3}({v}_{qg}{i}_{dg}-{v}_{dg}{i}_{qg})$$

#### Rotor side converter control

**Fig. 6:**
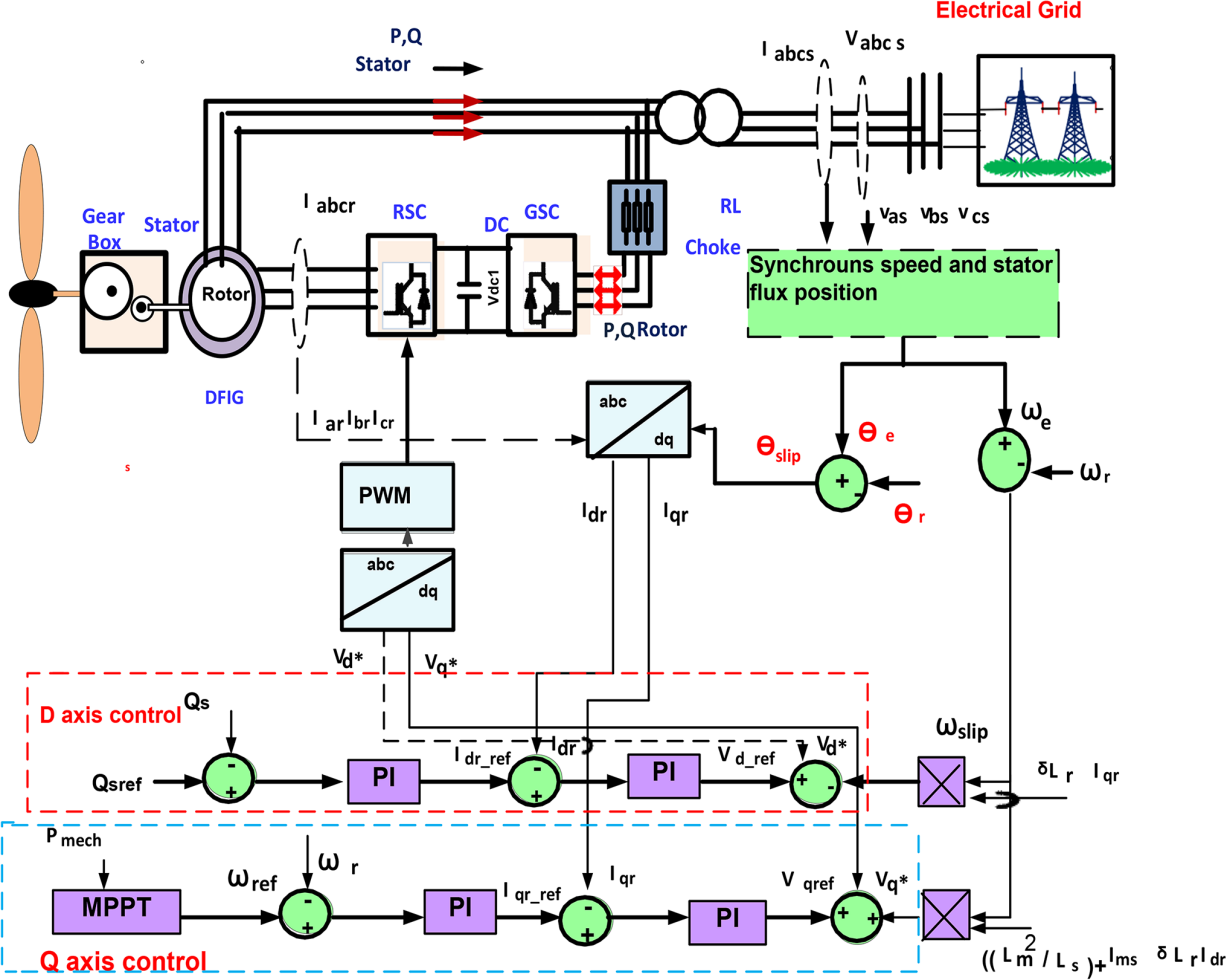
Control configuration of rotor side converter.

Figure 6 shows the control circuit of RSC. The active power of the stator, Ps, and electromagnetic torque, T_e_ was controlled during i_qr_, while the reactive power of the stator, Qs was detected by the i_dr_ as expressed in the following Equations^26,27^:

15 $$\:{v}_{dr-ref}={v*}_{dr}-\left({\omega\:}_{e}-{\omega\:}_{r}\right){\delta\:}_{s}{L}_{r}{i}_{qr}$$

16 $$v_{{qr - ref}} = v*_{{qr}} - \left( {\omega \:_{e} - \omega \:_{r} } \right)\left( {\delta _{s} L_{r} i_{{dr}} + \frac{{L_{m}^{2} }}{{L_{s} }}i_{{ms}} } \right)$$

## Fuzzy PI control

**Fig. 7:**
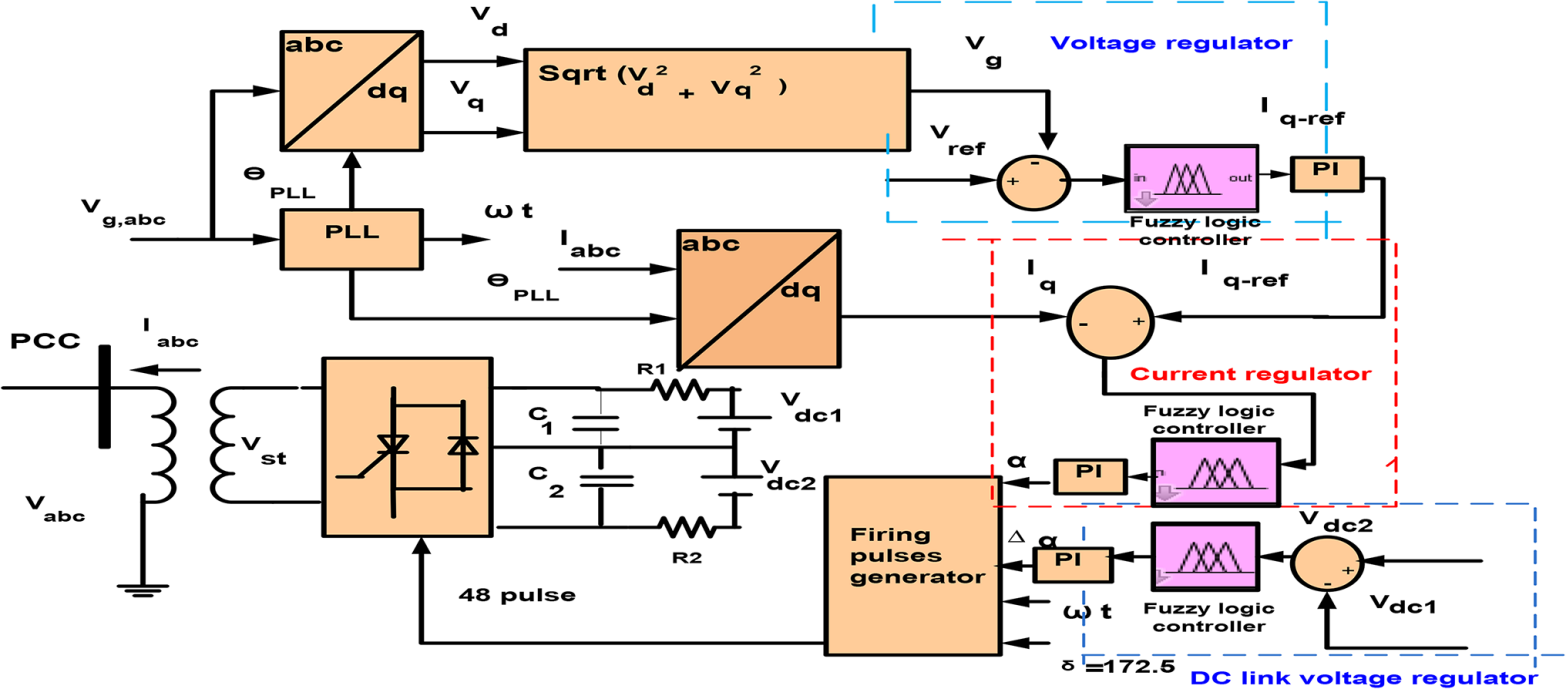
Configuration STATCOM control with PI Fuzzy logic controller.

**Fig. 8:**
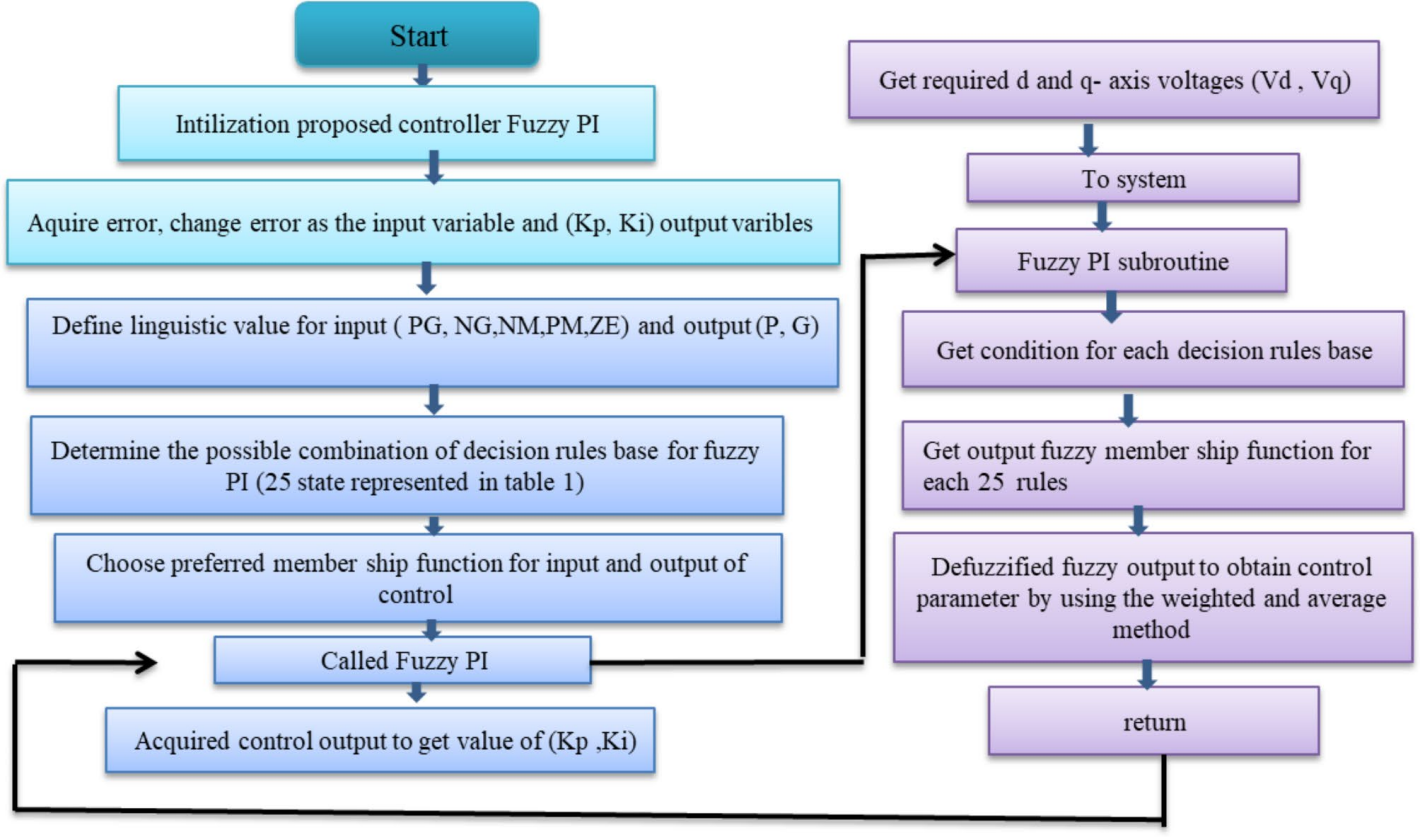
Flowchart of Fuzzy PI.

**Fig. 9:**
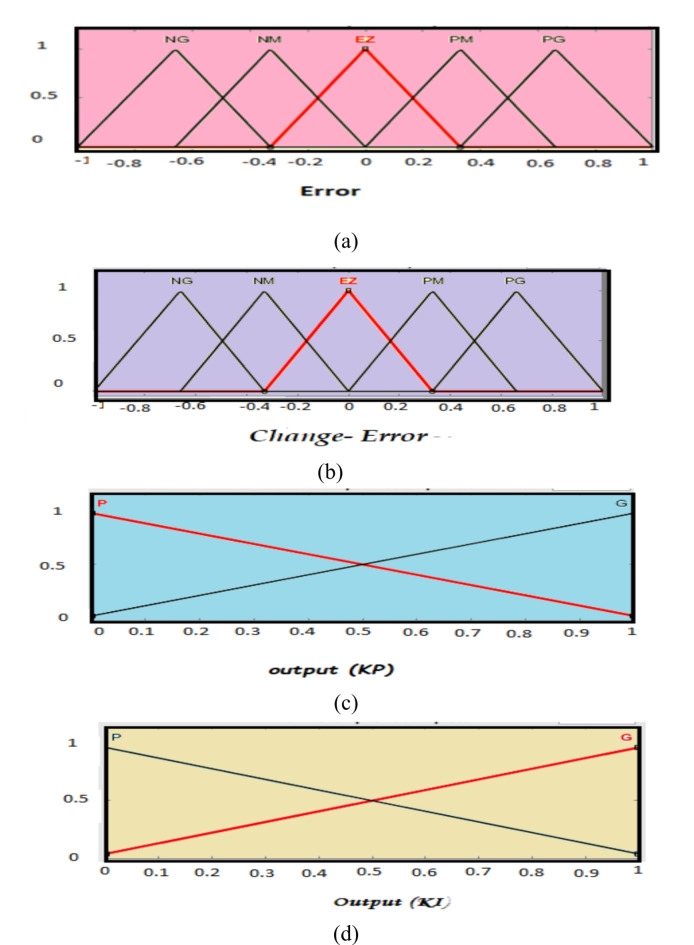
(**a**) Variable Input error. (**b**) Variable Input change-error. (**c**) Variable Output (K_p_), (**d**) variable Output (K_i_).

**Fig. 10:**
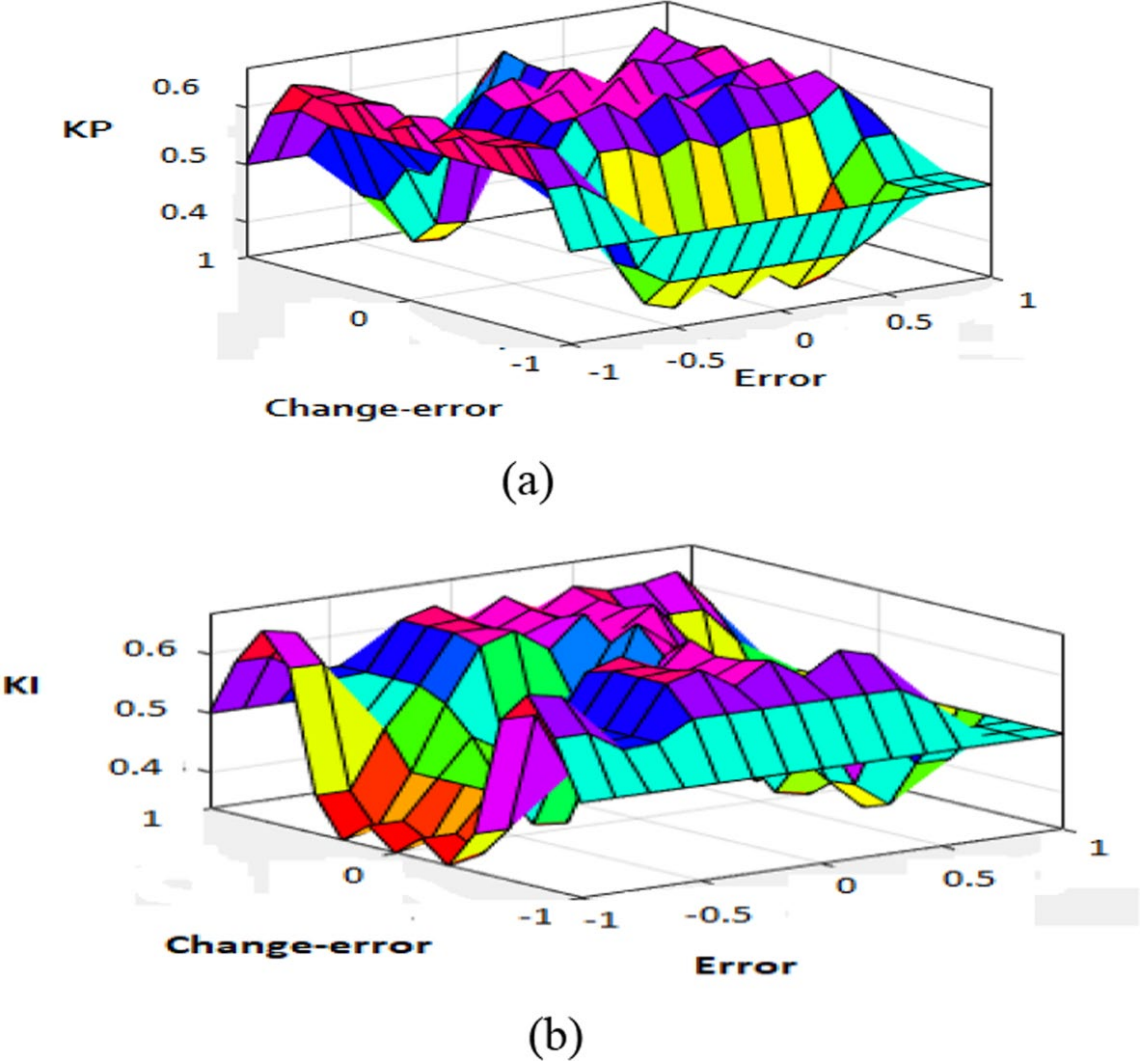
(**a**) Surface view of K_p_ of PI FLC, (**b**) surface view of K_i_ of PI FLC.

Figure 7 shows Fuzzy PI (FPI) control that is used in the simulation. FPI control has two inputs and three outputs. Where the error signal of d and q-axis and derivate of these errors are inputs of FPI, and K_p_, K_i_ are the outputs of FPI control. FPI control output used to obtain the required voltage (V_d_, V_q_) that are transformed to three-phase voltage V_abc_ by utilization to inverse park transformation. Figure 8 shows a flowchart of FPI, Table 4 shows the knowledge that represents in the shape of rules and Fig. 9a–d shows the membership function for input and output variables, and Fig. 10a, b shows surface view of K_p_ of PI FLC and surface view of K_i_ of PI FLC^27,48^.

**Table 4:** Rule base for fuzzy PI logic control.

Error	Change error									
	NG		NM		EZ		PM		PG	
EZ	Kp	Ki	Kp	Ki	Kp	Ki	Kp	Ki	Kp	Ki
	G	G	G	G	G	G	G	G	G	G
NG	Kp	Ki	Kp	Ki	Kp	Ki	Kp	Ki	Kp	Ki
	G	G	G	P	G	P	G	P	G	G
NM	Kp	Ki	Kp	Ki	Kp	Ki	Kp	Ki	Kp	Ki
	p	G	G	G	G	P	G	G	G	p
PM	Kp	Ki	Kp	Ki	Kp	Ki	Kp	Ki	Kp	Ki
	p	G	G	G	G	P	G	G	G	p
PM	Kp	Ki	Kp	Ki	Kp	Ki	Ki	Ki	Kp	Ki
	G	G	G	P	G	p	G	P	G	G

## LAPO optimization

**Fig. 11:**
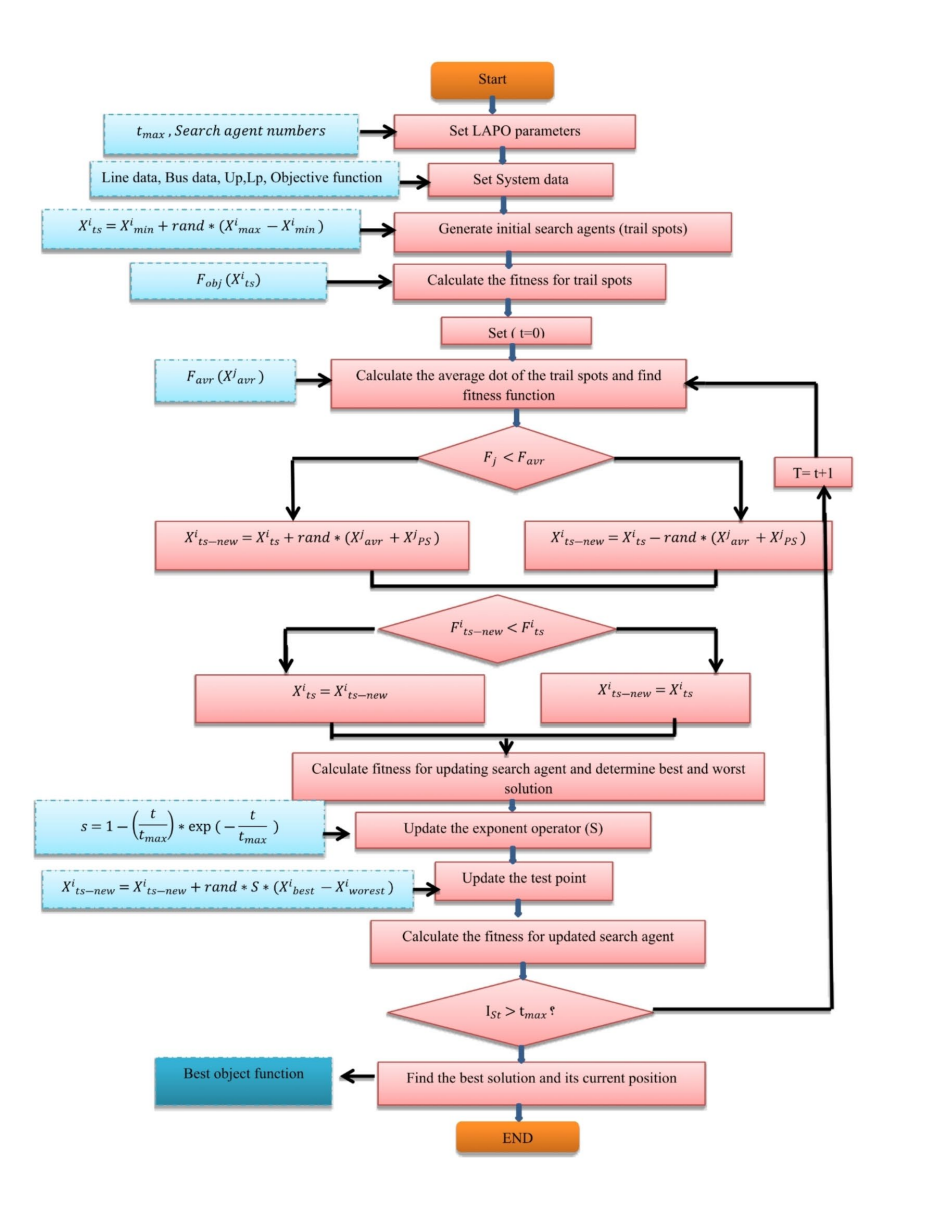
Flowchart of LAPO optimization.

The Lightning Attachment Procedure Optimization Algorithm is called (LAPO). It was established on the phenomenon of lightning and thunder^20^. LAPO is a new optimization method that took the form of the phenomena of Lightning where large amounts of electrical charges were accumulated in the cloud. Lightning was formed with a rising number of electrical charges on the form cloud that causes rising electrical strength so lightning strikes will happen and emit at various dots^49^. The LAPO optimization phenomenon consists of several steps. The first step is the conveyance charge of clouds to the ground. The second step is composing a flash. Composing thunder and lightning is the last step. The method of LAPO optimization is composed of population initialization, branch fading, upward leader movement, downward leader movement, and improvement of execution. The initialization of the population is composed of a group of dots, each dot acts as a member of the population this leads to discharge may happen. Leader of Downward motion acts random selection to choose the test dots from members. The Leader of Upward motion is the procedure of selecting the best and worst dot that has been generated by the Leader of downward motion. The branch fading could be characterized as coordination lightning by testing a dot that results from a leader of upward motion. This operation will be reiterated for each production^50,51^. Figure 11 shows the flowchart that introduces the LAPO optimization method, and Table 5 shows the parameter of FPI.

**Table 5:** LAPO parameters of control STATCOM.

LAPO parameters	Parameter of Voltage regulator		Parameter of current regulator		DC regulator	
	K_p_1	K_i_1	K_p_2	K_i_2	K_p_3	K_i_3
Three phase fault	13.6	2.8525e3	5.048	39.5	0.001	0.1
DLG	9.55	2.9086e3	3.129	41.5	0.0001	0.001

## Control circuit of STATCOM

STATCOM which produces 100 MVAR is linked to the network at a bus of PCC. The basic goal of STATCOM is to adjust the voltage of the PCC Bus by compensating reactive power. It forms of DC-link capacitor to compensate reactive power, coupling the transformer between the voltage source inverter and network and voltage source inverter that produces AC voltage. The Eqs. (17, 18) express active and reactive power transport between STATCOM and the electrical network^52–56^:

17 $$\:{\text{P}}_{\text{g}}=3\left(\frac{{\text{V}}_{\text{g}}{\text{V}}_{\text{s}\text{t}}}{{\text{x}}_{\text{t}\text{r}}}\right)\text{s}\text{i}\text{n}\left({\upalpha\:}\right)$$

18 $$\:{\text{Q}}_{\text{g}}=3\left(\frac{{\text{V}}_{\text{g}}{\text{V}}_{\text{s}\text{t}}}{{\text{x}}_{\text{t}\text{r}}}\right)\text{cos}\left({\upalpha\:}\right)-\frac{{\text{V}}_{\text{g}}^{2}}{{\text{x}}_{\text{t}\text{r}}}$$

**Fig. 12:**
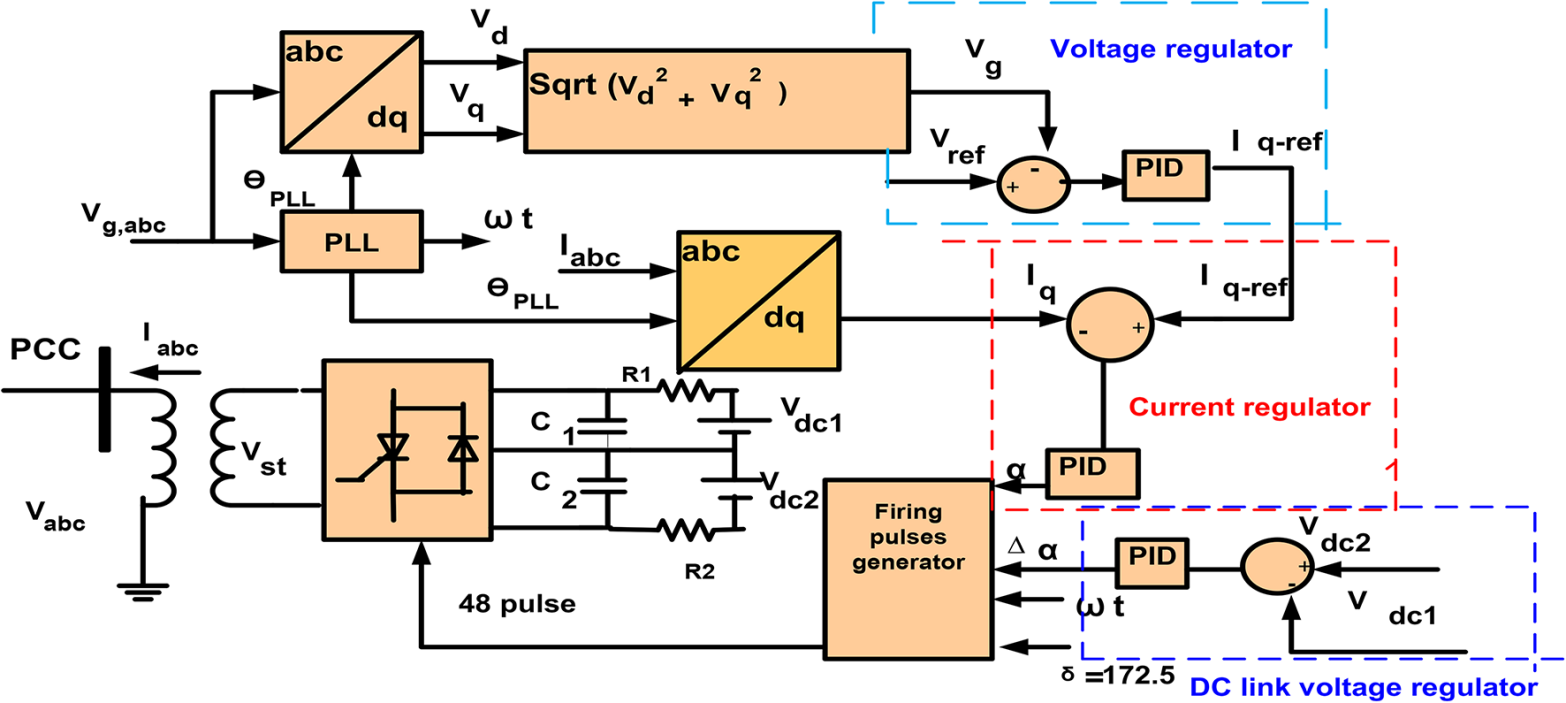
Configuration STATCOM control with PID.

Figure 12 shows the control circuit of STATCOM with PID control. It is composed of PLL that produces $\:{{\uptheta\:}}_{PLL}$ that called the reference angle for the transformation of abc to dq. In a loop of voltage regulation, effective voltage, V_g_ of the PCC Bus is contrasted with V_ref_ called reference voltage and variance is applied to PID control to produce I_q−ref_ that called reference reactive current. Also, in a loop of current regulator contrasts compensated I_q_ that called reactive current with I_q−ref_ that called reference value to generate desired phase angle, α. Where α is a phase shift of STATCOM voltage, V_st_ relative to the voltage of grid V_g_^53^. σ is called the angle of conduction for three-level inverters and is reinforced to 172.5° to reduce the harmonics of the 23rd and 25th. Also, the DC-link voltage regulator was used to reduce harmonics, and the voltage negative and positive of the DC capacitor was compelled to preserve equality by using ∆α called slight offset from the DC voltage regulator^21,51–56^. Controlling of STATCOM is shown by the following equations:

19 $$\:{\text{I}}_{\text{q}-\text{r}\text{e}\text{f}}=({\text{V}}_{\text{r}\text{e}\text{f}}-{\text{V}}_{g})\left(\:\frac{{{\text{K}}_{\text{D}}\text{S}}_{\:}^{\:2}+{\text{K}}_{\text{P}}\text{s}+\:{\text{K}}_{\text{I}}}{\text{S}}\right)$$

20 $$\:{\upalpha\:}=({\text{I}}_{\text{q}-\text{r}\text{e}\text{f}}+{\text{I}}_{\text{q}})\left(\:\frac{{{\text{K}}_{\text{D}}\text{S}}_{\:}^{\:2}+{\text{K}}_{\text{P}}\text{s}+\:{\text{K}}_{\text{I}}}{\text{S}}\right)$$

21 $$\:{\Delta\:}{\upalpha\:}=({\text{V}}_{\text{d}\text{c}2}-{\text{V}}_{\text{d}\text{c}1})\left(\:\frac{{{\text{K}}_{\text{D}}\text{S}}_{\:}^{\:2}+{\text{K}}_{\text{P}}\text{s}+\:{\text{K}}_{\text{I}}}{\text{S}}\right)$$

## Simulation results and discussion

Figure 1 illustrates the Simulink model of an electrical grid connected to a PV/wind hybrid system that generates active power equal to 10 MW divided into 9 MW from the wind farm and 1 MW from the PV station integrated with 100 MVAR STATCOM and energizes load with 9.2 MW. During natural operating conditions, the rated wind speed of the wind farm is (12 m/s) and the PV system operating conditions are irradiance, G = 1000 W/m^2^, and temperature, T = 25 °C. Various types of faults such as three-phase faults and double lines to the ground are studied to know the effect of LVRT on electrical grid and hybrid power systems. Also, comparisons between different types of control such as PID controller, and PI Fuzzy logic controller are studied to mitigate the effect of LVRT and improve voltage stability.

### Case-1: three phase fault

Three-phase fault is applied at 0.5 s and cleared at 0.7 s in the electrical grid at the bus (120 KV).The fault affects wind bus 575 KV, bus 25 KV and also affects PV station. Voltage sag occurs on the voltage of wind bus and PV bus and also affects active power, reactive power reducing and voltage of the DC link of the wind and PV. So STATCOM 100 MVAR is connected to mitigate voltage sag, compensate active and reactive power of wind turbine and reduce oscillations of DC link voltage.

#### DFIG model results

**Fig. 13:**
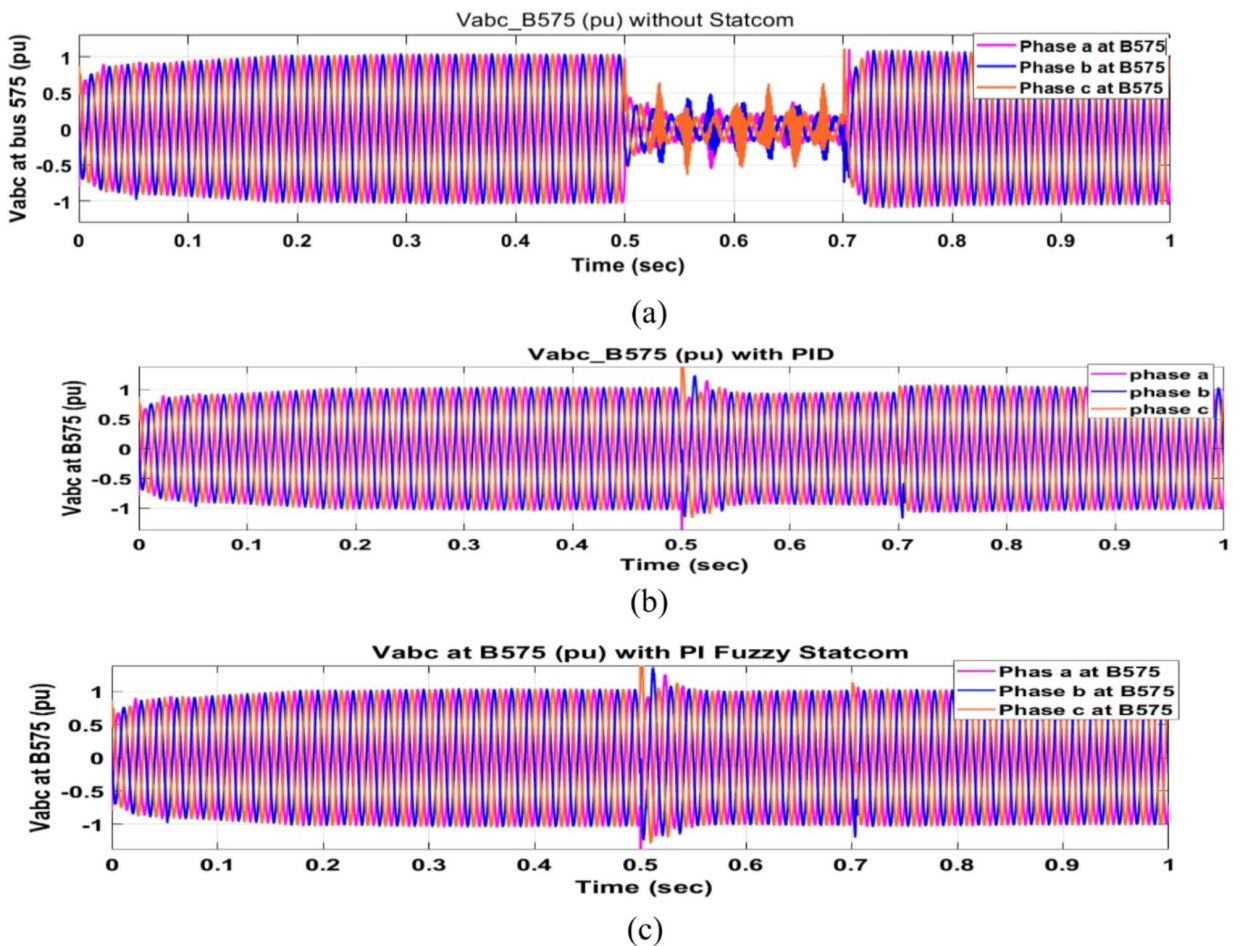
Voltage at Bus 575 during three-phase fault. (**a**) Voltage at Bus 575 during three-phase fault without connects STATCOM. (**b**) Voltage at Bus 575 with PID controller STATCOM. (**c**) Voltage at Bus 575 with PI Fuzzy STATCOM.

**Fig. 14:**
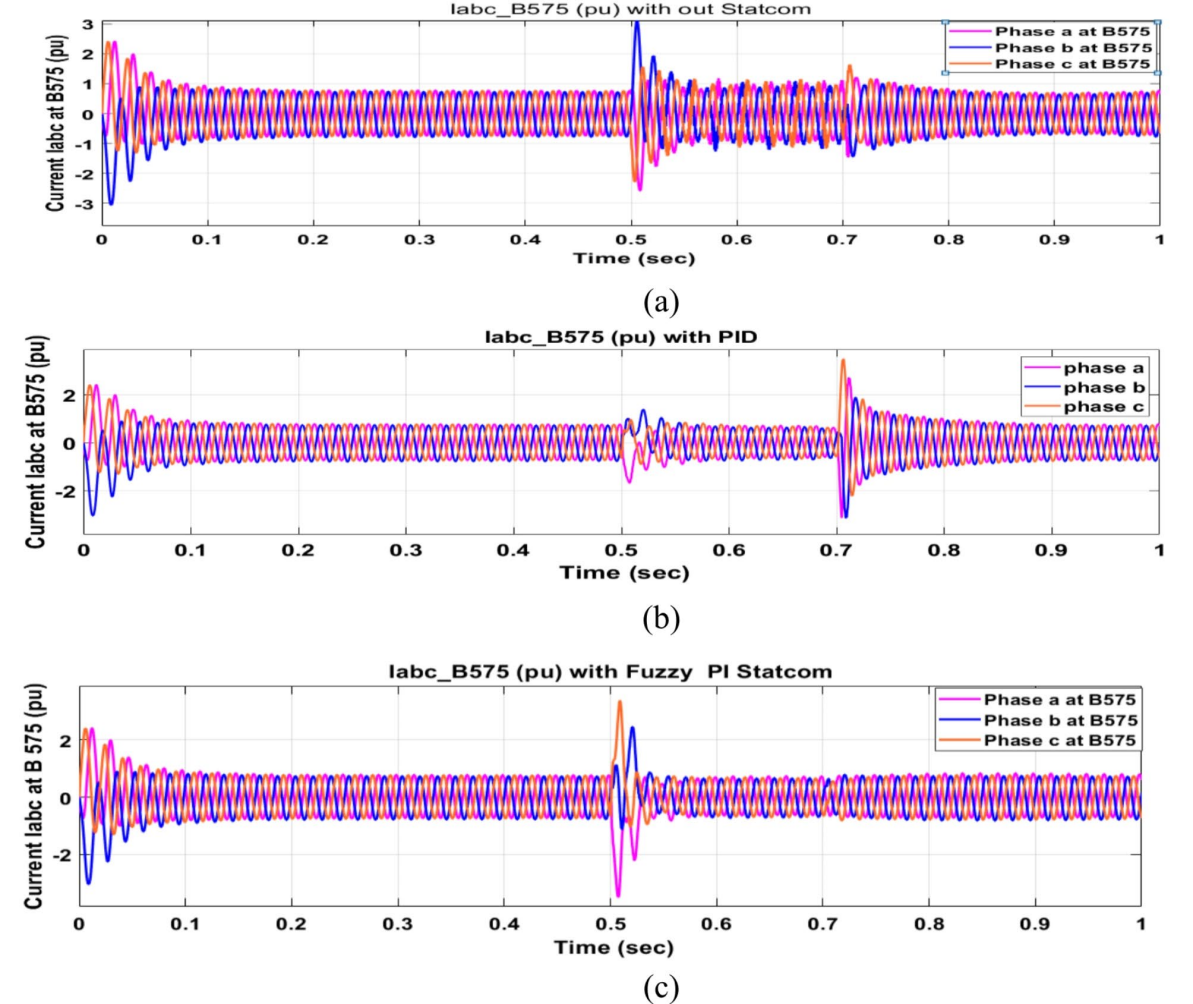
Current at Bus 575 during three-phase fault. (**a**) Current at Bus 575 during three-phase fault without connects STATCOM. (**b**) Current at Bus 575 with PID controller STATCOM. (**c**) Current at Bus 575 with PI Fuzzy control STATCOM.

**Fig. 15:**
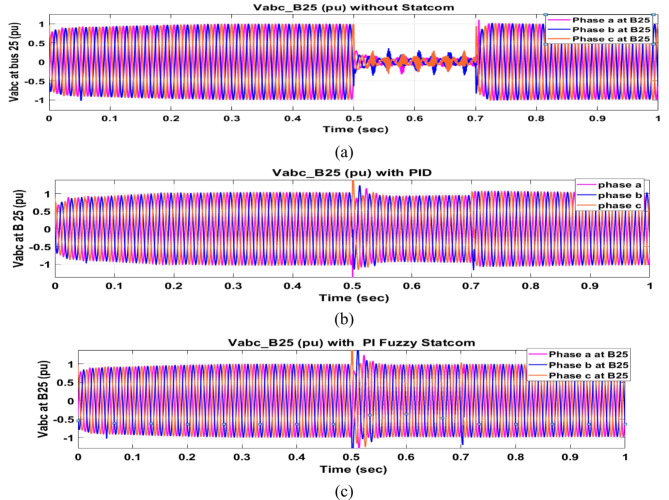
Voltage at Bus 25 during three phase fault. (**a**) Voltage at Bus 25 during three phase fault without connects STATCOM. (**b**) Voltage at Bus 25 with PID controller STATCOM. (**c**) Voltage at Bus 25 with PI Fuzzy control. STATCOM.

**Fig. 16:**
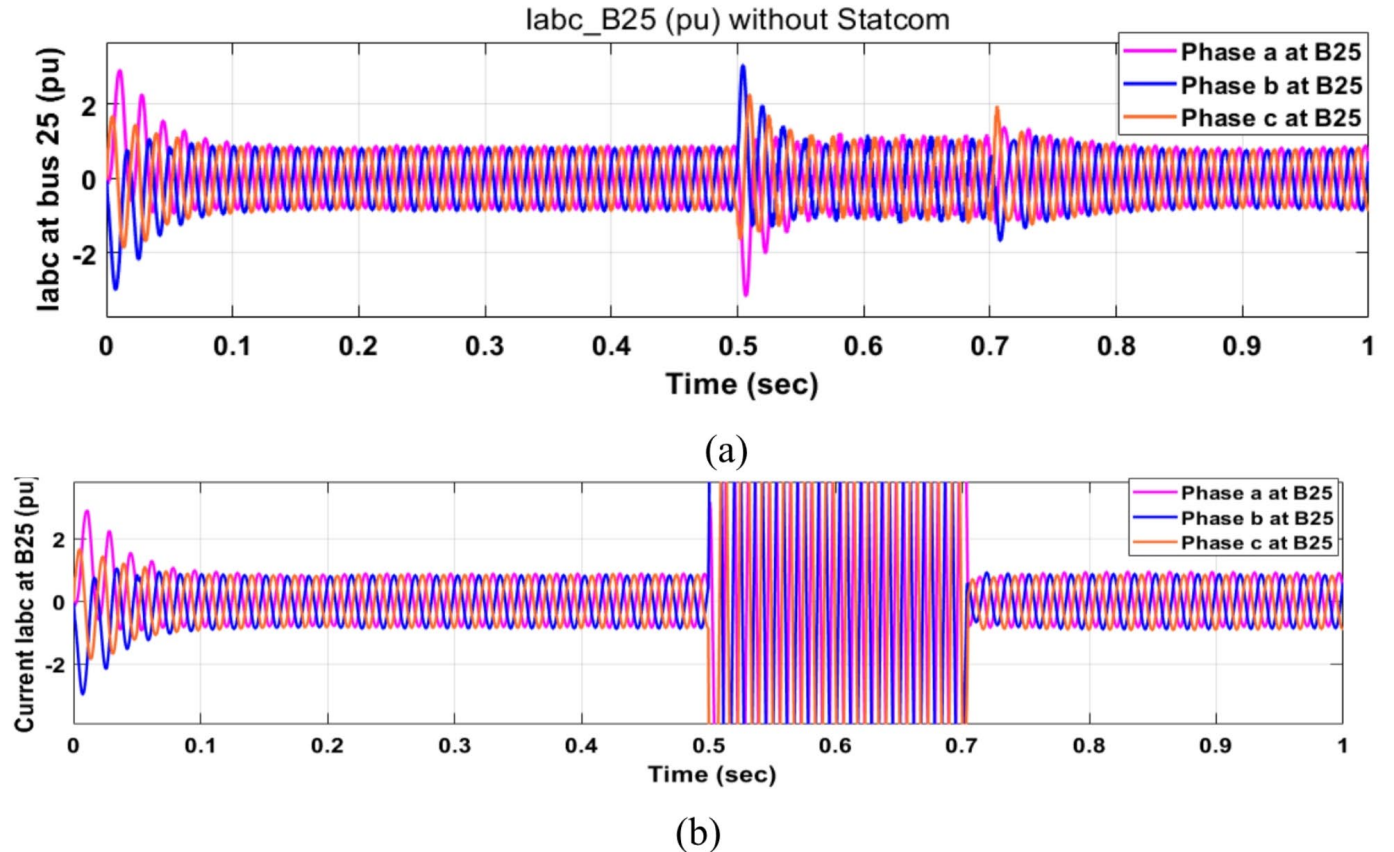
Current at Bus 25 during three-phase fault. (**a**) Current at Bus 25 during three-phase fault without connects STATCOM. (**b**) Current at Bus 25 with PID controller STATCOM, and current at Bus 25 with PI Fuzzy control STATCOM.

**Fig. 17:**
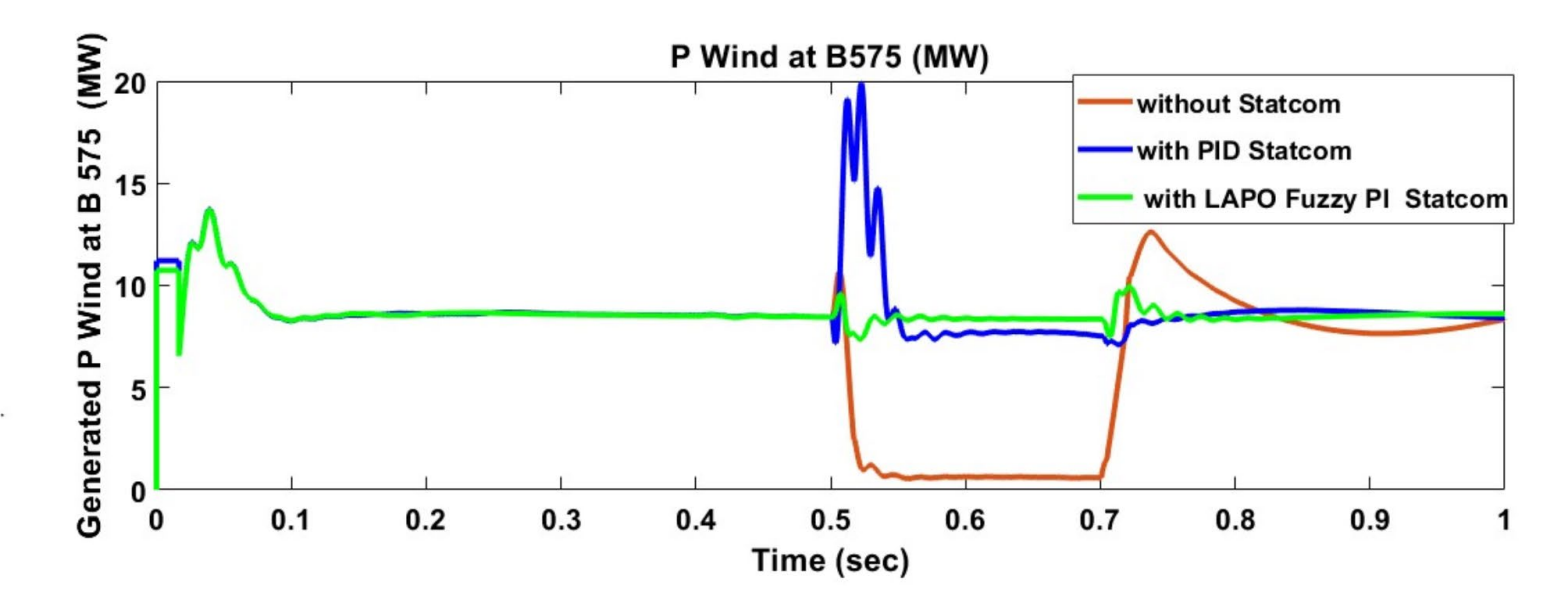
Generated active power at Bus 575 during three-phase fault without connects STATCOM, with PID controller STATCOM, and with PI Fuzzy control STATCOM.

**Fig. 18:**
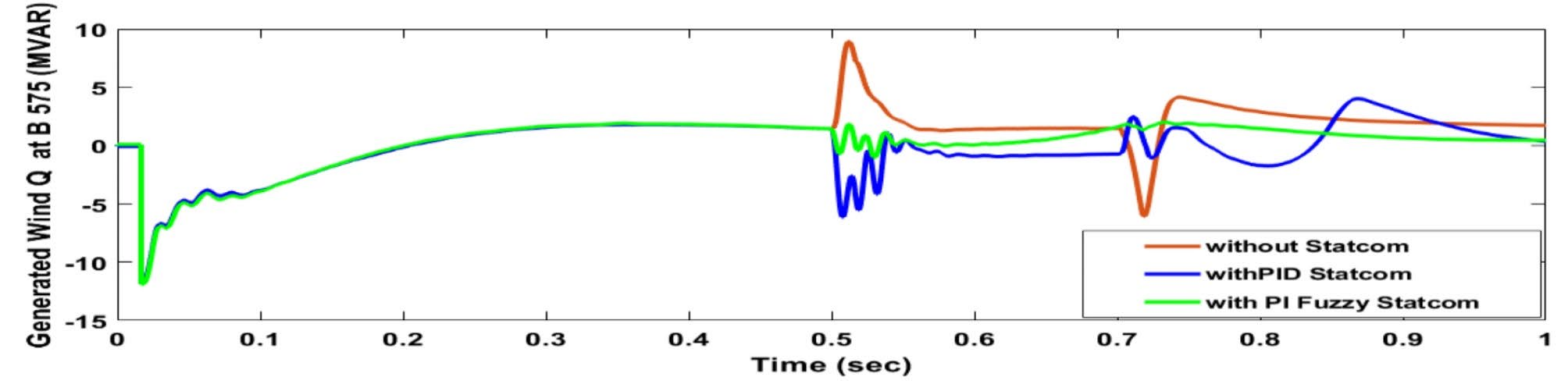
Generated reactive power at Bus 575 through three-phase fault without connects STATCOM, with PID controller STATCOM, and with PI Fuzzy control STATCOM.

**Fig. 19:**
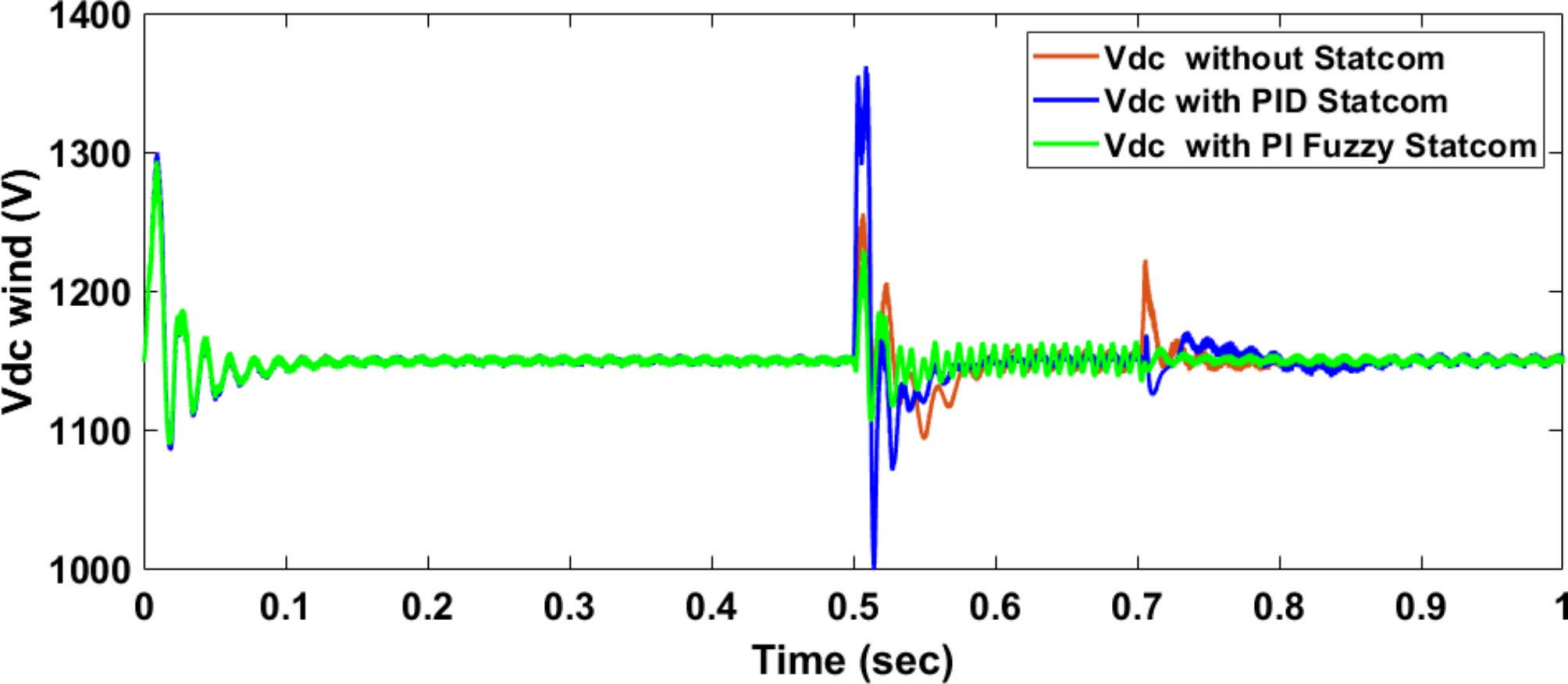
Voltage of DC link at Bus 575 through three-phase fault without connects STATCOM, with PID controller STATCOM, and with PI Fuzzy control STATCOM.

Voltage sag occurred at bus 575 V was reduced at the period of fault as shown in Fig. 13a and when PID and FPI STATCOM connected voltage sag was mitigated as shown in Fig. 13b, c. Also, Fig. 14a–c shows the current before and after connected STATCOM to mitigate voltage swell. Voltage sag occurred at bus 25 KV was reduced, when STATCOM was not connected as shown in Fig. 15a and mitigated when STATCOM connected as shown in Fig. 15b, c. Figure 16a, b shows current at bus 25 before and after the connected STATCOM current swell occurred but STATCOM does not mitigate swell in current at bus 25 KV. Figure 17 shows active power before and after connecting STATCOM with PID and FPI. DFIG reactive power Q = 0. Figure 18 shows reactive power before and after connecting STATCOM with PID and FPI. Figure 19 shows the voltage of the DC link before and after linking STATCOM with PID and FPI. Comparisons between the PID controller and PI Fuzzy logic controller are studied to improve LVRT as shown in Table 6. Also, the time domain analysis of the DC link voltage for the wind bus is shown in Table 7.

**Table 6:** Comparisons of the results through three-phase fault at B 575 V.

Comparison cases	Active power (MW) minimum–maximum	Reactive power (MVAR) minimum–maximum
Without STATCOM	0.6–12.55	− 5.87–8.9
With PID STATCOM	7.3–19.8	− 6.1–2.35
With PI Fuzzy STATCOM	8.5–9	− 0.11–1.27

**Table 7:** The time domain analysis for DC–link voltage through three-phase fault at wind bus.

At t = 0.5 s				At t = 0.7 s		
Comparison cases	Settling time	Under shoot	Over shoot	Settling time	Under shoot	Over shoot
Without STATCOM	0.5866	1096.02	1254.79	0.7867	1142.46	1221.69
With PID STATCOM	0.5629	1001.23	1361.86	0.8422	1128.77	1166.99
With PI Fuzzy STATCOM	0.57928	1110.47	1224.81	0.75121	1137.3	1161.96

#### PV model results

**Fig. 20:**
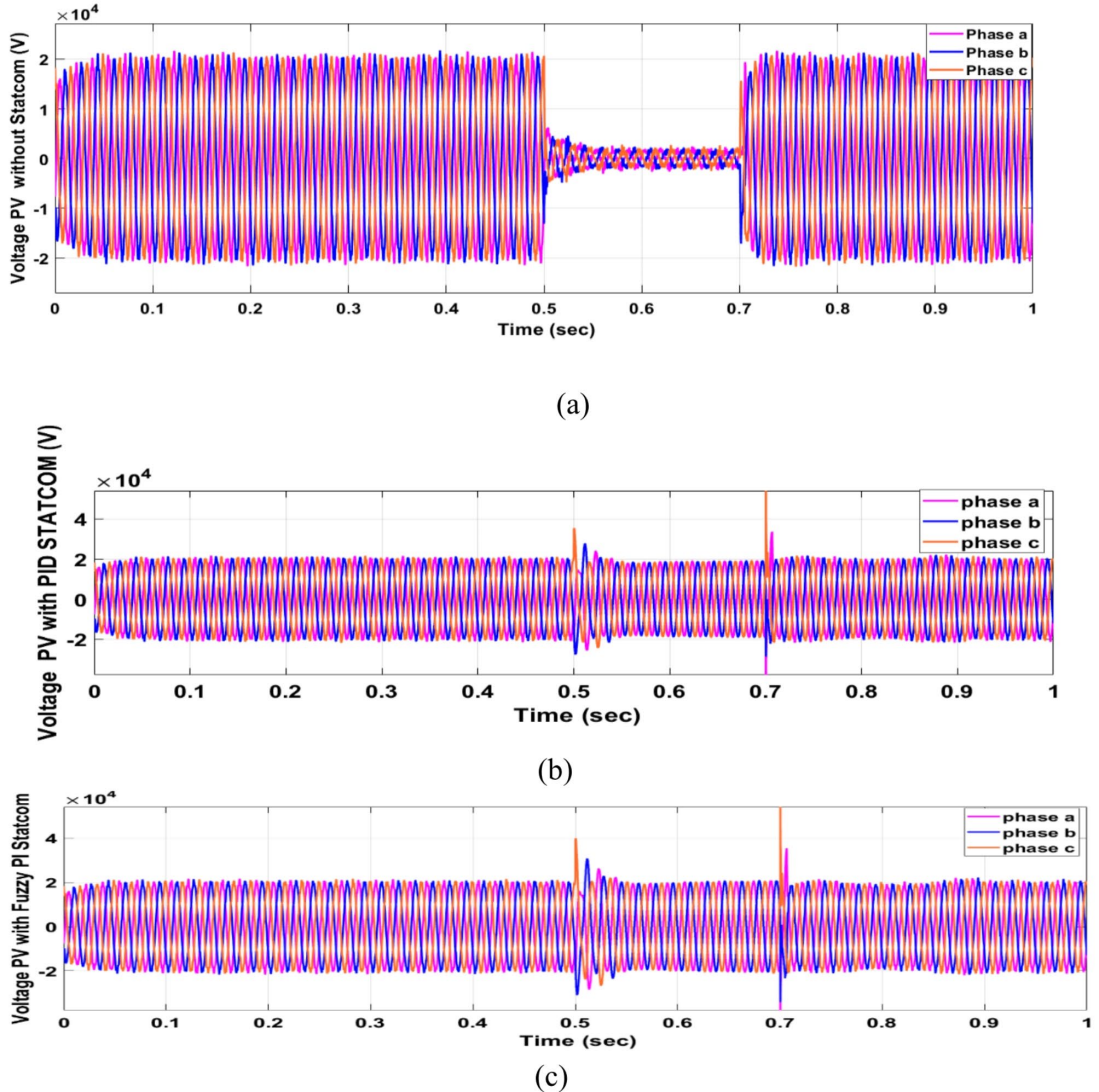
Voltage at PV bus during three-phase fault. (**a**) Voltage at PV Bus during three-phase fault without connects STATCOM. (**b**) Voltage at PV Bus with PID controller STATCOM. (**c**) Voltage at PV Bus with PI Fuzzy control STATCOM.

**Fig. 21:**
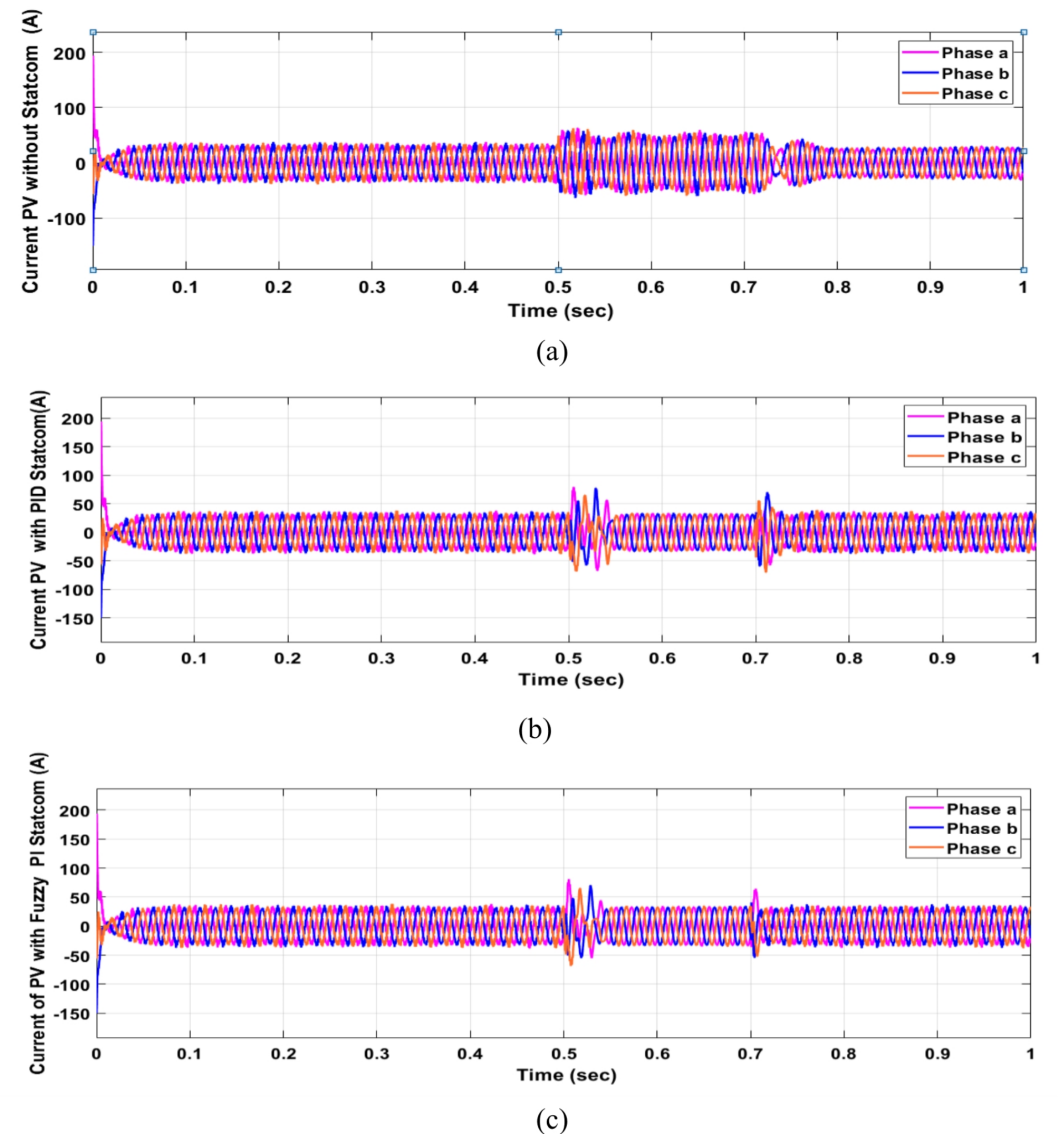
Current PV Bus during three-phase fault. (**a**) Current PV Bus during three-phase fault without connects STATCOM. (**b**) Current at PV Bus with PID controller STATCOM. (**c**) Current at PV Bus with PI Fuzzy control STATCOM.

**Fig. 22:**
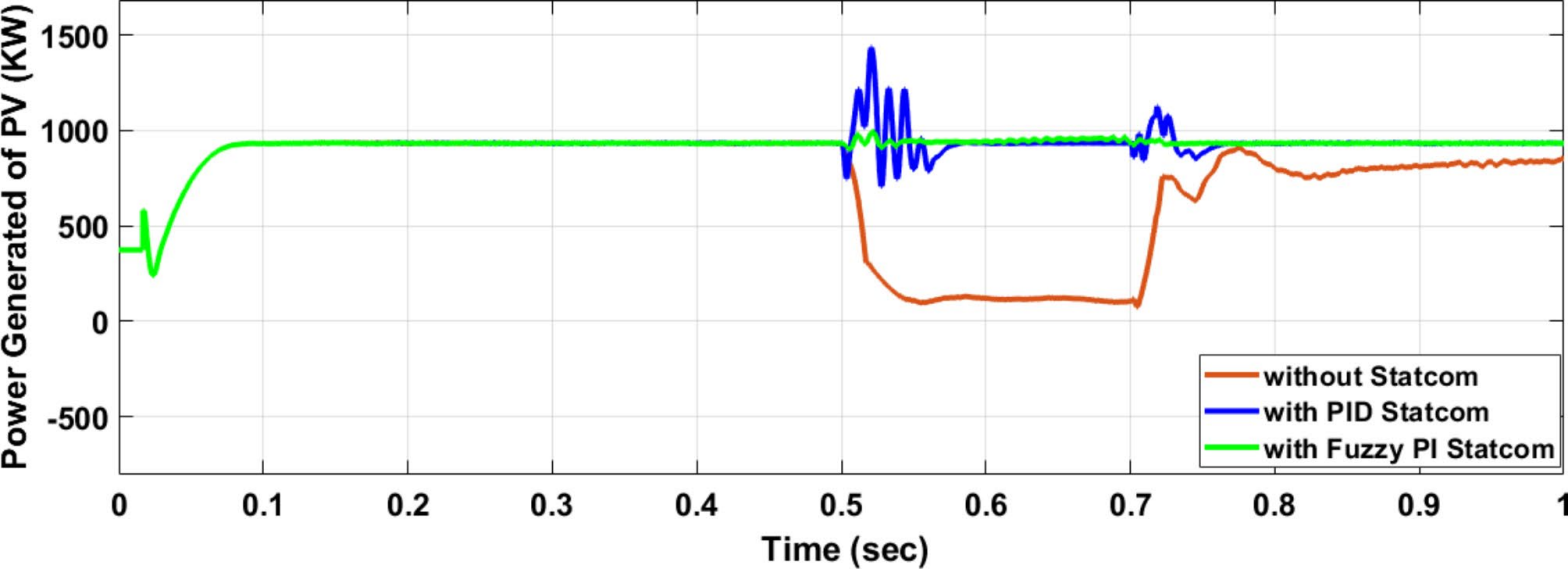
Generated active power at PV Bus during three-phase fault without connect STATCOM, with PID controller STATCOM, and with PI Fuzzy control STATCOM.

**Fig. 23:**
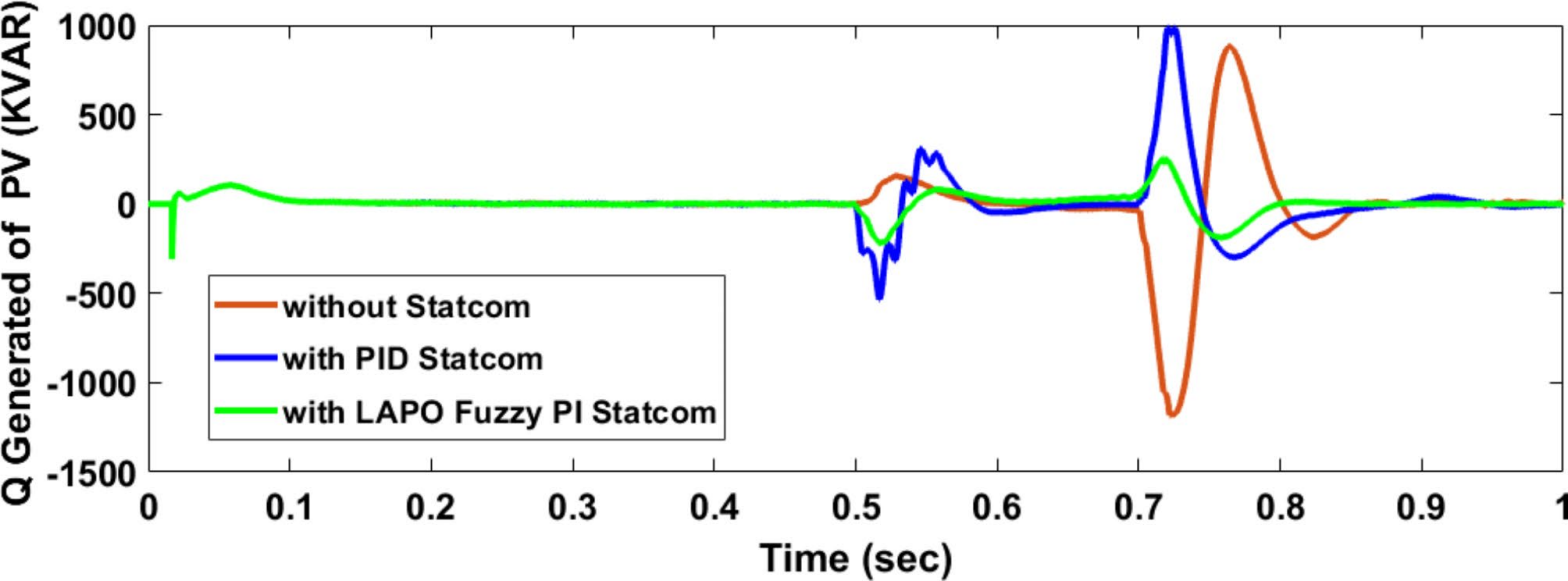
Generated reactive power at Bus 25 during three-phase fault without connects STATCOM, with PID controller STATCOM, and with PI Fuzzy control STATCOM.

**Fig. 24:**
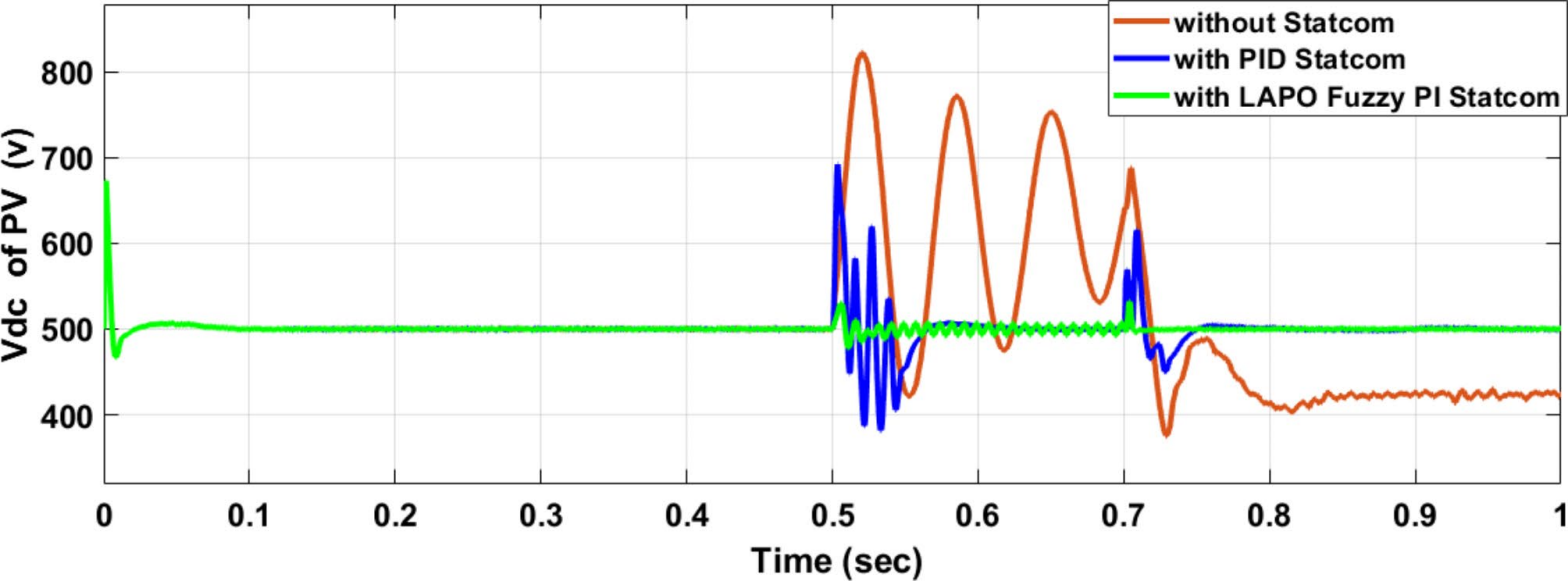
Voltage PV of DC link during three-phase fault without connect STATCOM, with PID controller STATCOM, and with PI Fuzzy control STATCOM.

Voltage sag occurred at PV bus voltage was reduced to at the period of fault as shown in Fig. 20a and when PID and FPI STATCOM connected voltage sag was mitigated as shown in Fig. 20b, c. Also, Fig. 21a–c shows the current before and after connected STATCOM to mitigate voltage swell. Figure 22 shows active power before and after connecting STATCOM with PID and FPI. PV reactive power Q = 0. Figure 23 shows reactive power before and after connecting STATCOM with PID and FPI. Figure 24 shows the DC Link voltage before and after connecting STATCOM with PID and FPI. Comparisons between the PID controller and PI Fuzzy logic controller are studied to improve LVRT as shown in Table 8. Also, the time domain analysis of the DC link voltage for the PV bus is shown in Table 9.

**Table 8:** Comparisons of results through three-phase fault at PV bus.

Comparison cases	Active power (KW) minimum–maximum	Reactive power (KVAR) minimum–maximum
Without STATCOM	127–757	− 1173–127.77
With PID STATCOM	714–1340	− 525–886
With PI Fuzzy STATCOM	910–972	− 135–184

**Table 9:** The time domain analysis for DC –link voltage through three-phase fault at PV bus.

At t = 0.5 s				At t = 0.7 s		
Comparison cases	Settling time	Under shoot	Over shoot	Settling time	Under shoot	Over shoot
Without STATCOM	0.83455	421.592	822.16	0.83455	375.33	681.62
With PID STATCOM	0.5609	386.76	689.539	0.7472	456.837	611.875
With PI Fuzzy STATCOM	0.5381	477.86	528.64	0.7172	496.208	524.259

#### Hybrid PV/wind and load

**Fig. 25:**
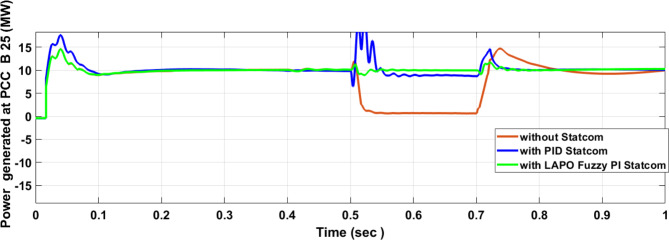
Active power generated at Bus 25 during three-phase fault without STATCOM, with PID STATCOM, and with Bus 25 with PI Fuzzy STATCOM.

**Fig. 26:**
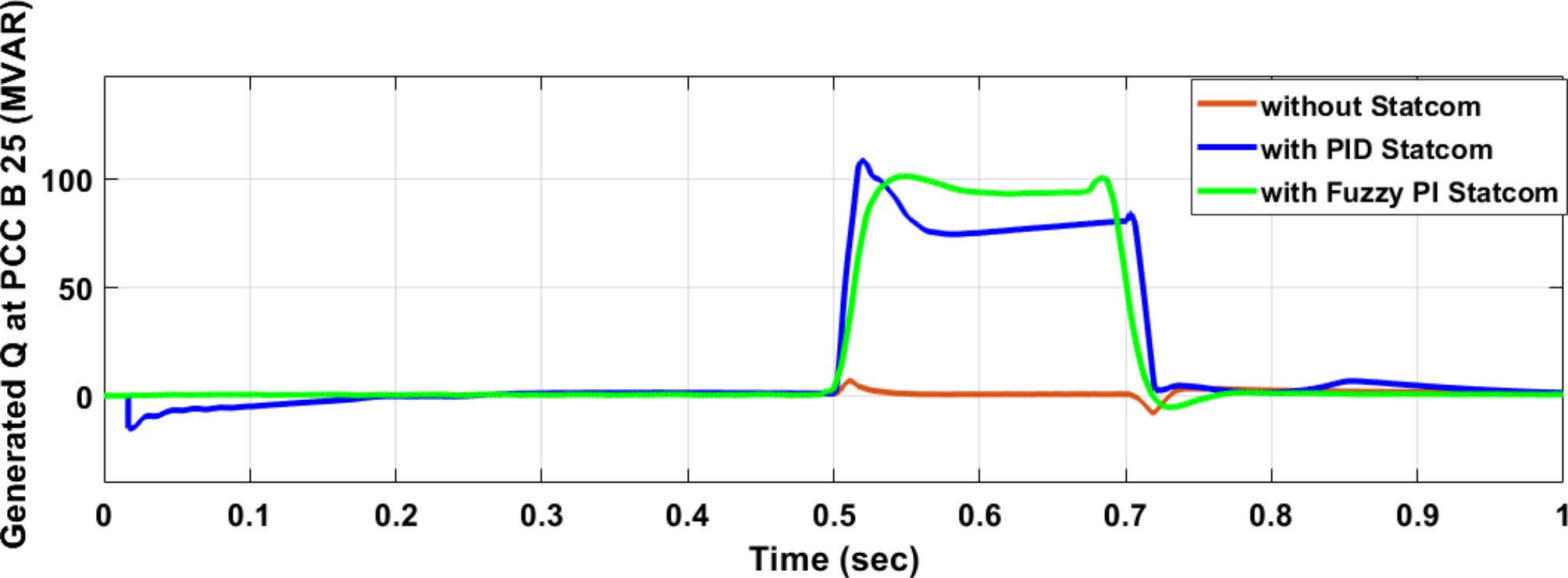
Reactive power generated at PCC Bus 25 during three-phase fault without STATCOM, with PID STATCOM, and with Bus 25 with LAPO PI Fuzzy STATCOM.

**Fig. 27:**
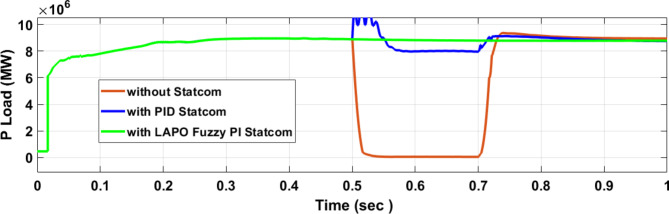
Load active power at Bus 25 during three-phase fault without STATCOM, with PID STATCOM, and with Bus 25 with PI Fuzzy STATCOM.

**Fig. 28:**
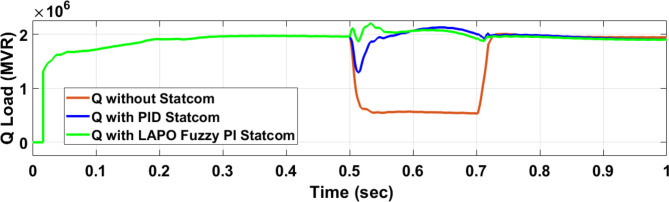
Load reactive power at Bus 25 during three-phase fault without STATCOM, with PID STATCOM, and with Bus 25 with PI Fuzzy STATCOM.

**Fig. 29:**
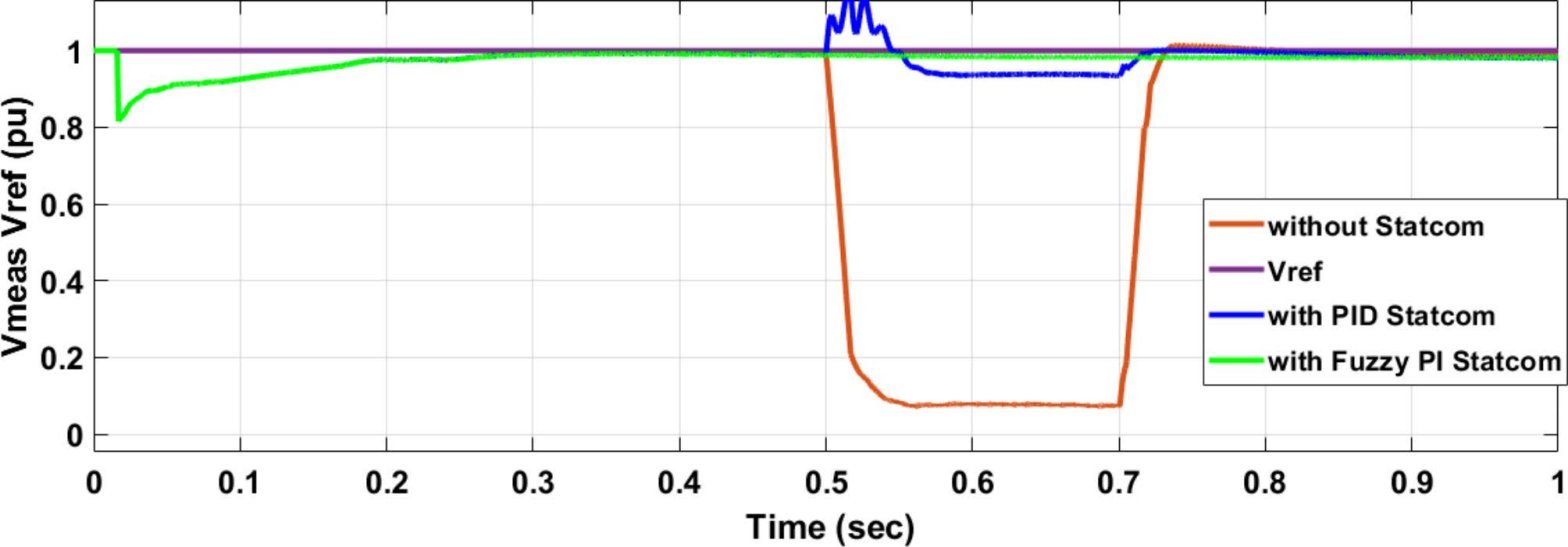
V_meas_, V_ref_ STATCOM during three-phase fault without connects STATCOM, Vref, with PID controller STATCOM, and with B 25 with PI Fuzzy control STATCOM.

**Fig. 30:**
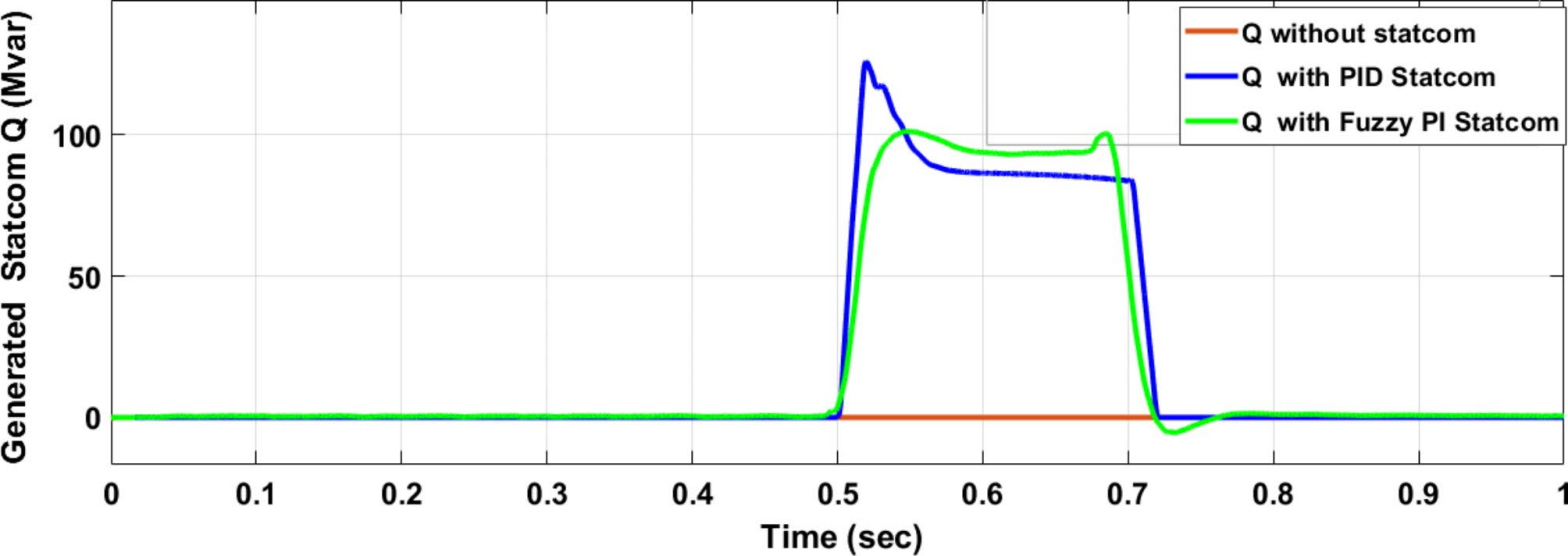
Reactive power generated during three-phase fault without connects STATCOM, with PID controller STATCOM, and with Bus 25 with PI Fuzzy control STATCOM.

Figure 25 shows the active power of hybrid PV/wind generated at Bus 25 before and after connected STATCOM in case PID, and FPI. Figure 26 shows reactive power before and after connecting STATCOM with PID, and FPI. Figure 27 shows the active power of load before and after connected STATCOM. Figure 28 shows the reactive power of load before and after connecting STATCOM with PID, and FPI. Figure 29 shows V_meas_, V_ref_ at bus STATCOM before and after STATCOM connected with PID and FPI. Also, Fig. 30 shows reactive power generated at bus STATCOM with PID, and FPI. Comparisons between the PID controller and PI Fuzzy logic controller are studied to improve LVRT as shown in Table 10. Also, Comparisons between the PID controller and PI Fuzzy logic controller are studied at STATCOM Bus to improve LVRT as shown in Table 11.

**Table 10:** Comparisons of results through three-phase fault at hybrid PV/wind at 25 KV Bus.

Comparison cases	Active power (MW) minimum–maximum	Reactive power	P Load	Q Load
Without STATCOM	0.7–13.4	0	0.05	0.67
With PID STATCOM	7.5–17.8	75.3	8	1.85
With PI Fuzzy STATCOM	10–10	95.8	9	1.96

**Table 11:** Comparisons of results through three-phase fault at STATCOM Bus.

Comparison cases	Voltage Vmeas (pu)	Vref (pu)	Reactive power
Without STATCOM	0.07	1	0
With PID STATCOM	0.936	1	86
With PI Fuzzy STATCOM	0.985	1	98.8

### Case 2: double line to ground fault (DLGF)

The DLGF is stratified at 0.5 s and cleared at 0.7 s in electrical grid fault effect on wind bus 575 V, bus 25 KV, and also effect on PV station. Voltage sag occurs on the voltage of wind bus and PV bus also affects active power, reactive power reducing, and DC link voltage of wind and PV. So, STATCOM 100 MVAR is connected to compensate voltage sag and compensate active and reactive power of wind turbine and reduce oscillations of DC link voltage as shown in Figs. 31, 32, 33, 34, 35, 36, 37, 38, 39, 40, 41, 42, 43, 44, 45, 46, 47, 48 and 49.

#### DFIG model

**Fig. 31:**
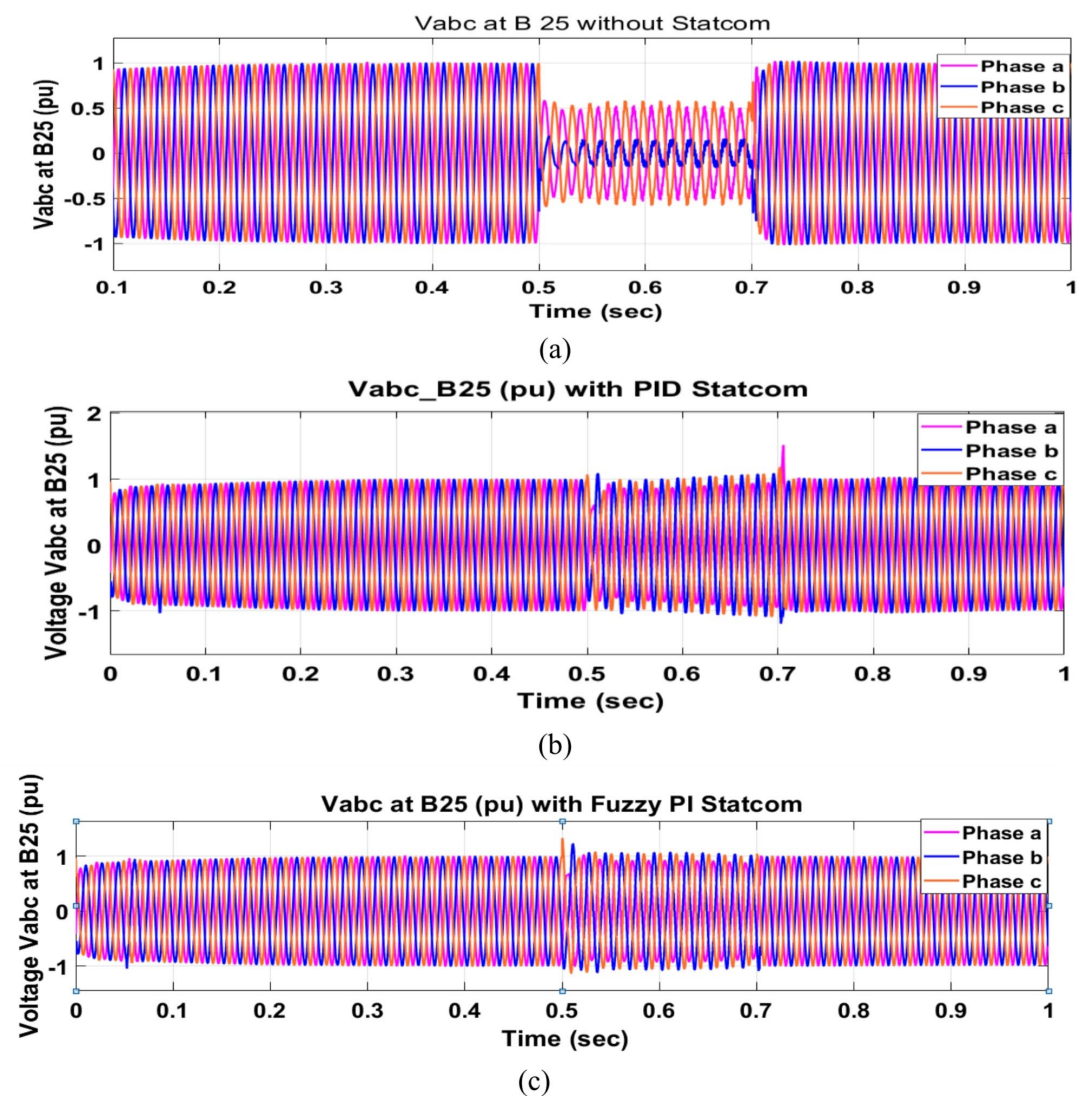
Voltage at Bus 25 through DLG. (**a**) Voltage at Bus 25 through DLG without connects STATCOM. (**b**) Voltage at Bus 25 with PID controller STATCOM and (**c**) Voltage at Bus 25 with PI Fuzzy control STATCOM.

**Fig. 32:**
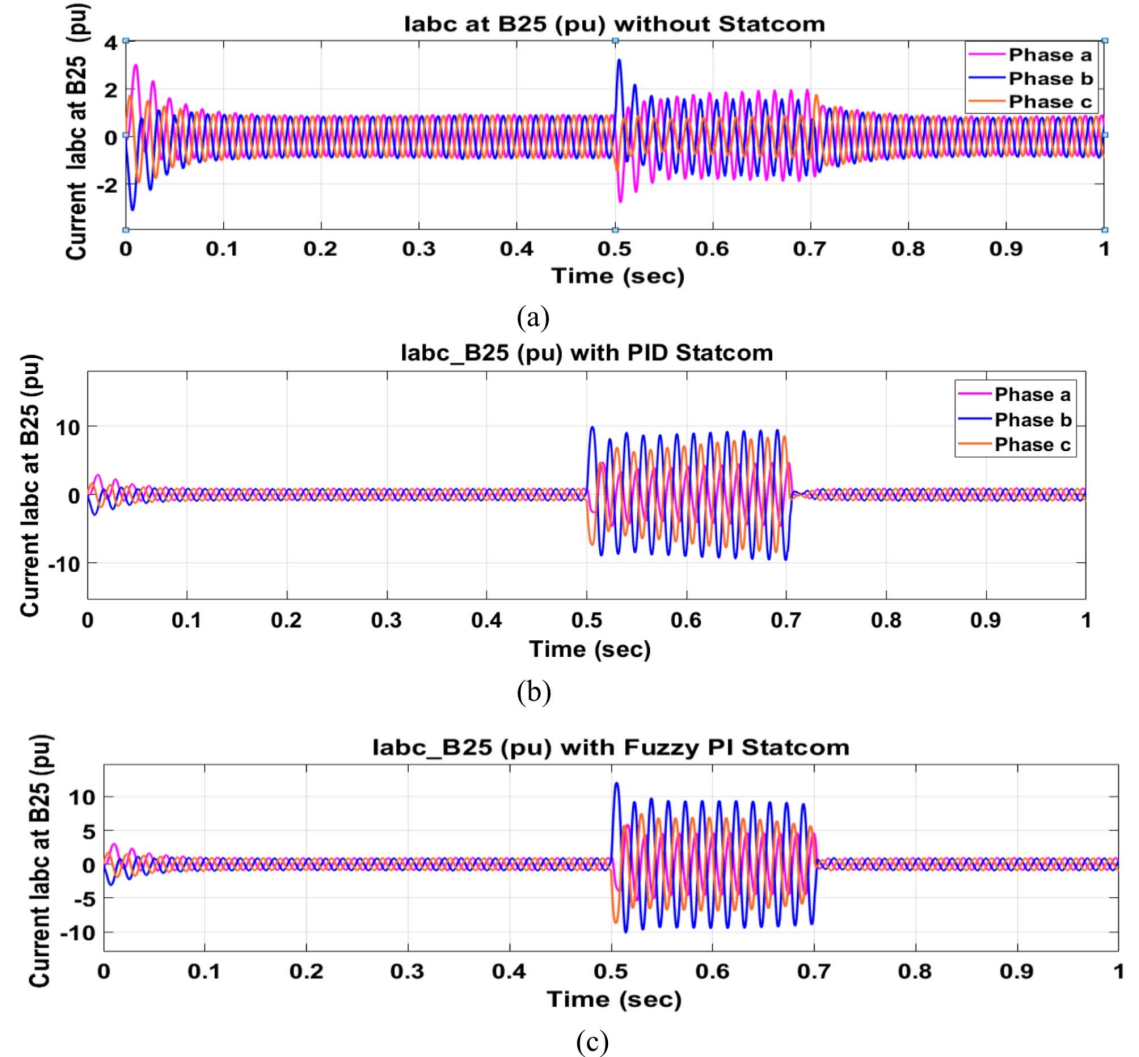
Current at Bus 25 through DLG. (**a**) Current at Bus 25 through DLG without connects STATCOM. (**b**) Current at Bus 25 through DLG with PID controller STATCOM. (**c**) Current at Bus 25 through DLG with PI Fuzzy control STATCOM.

**Fig. 33:**
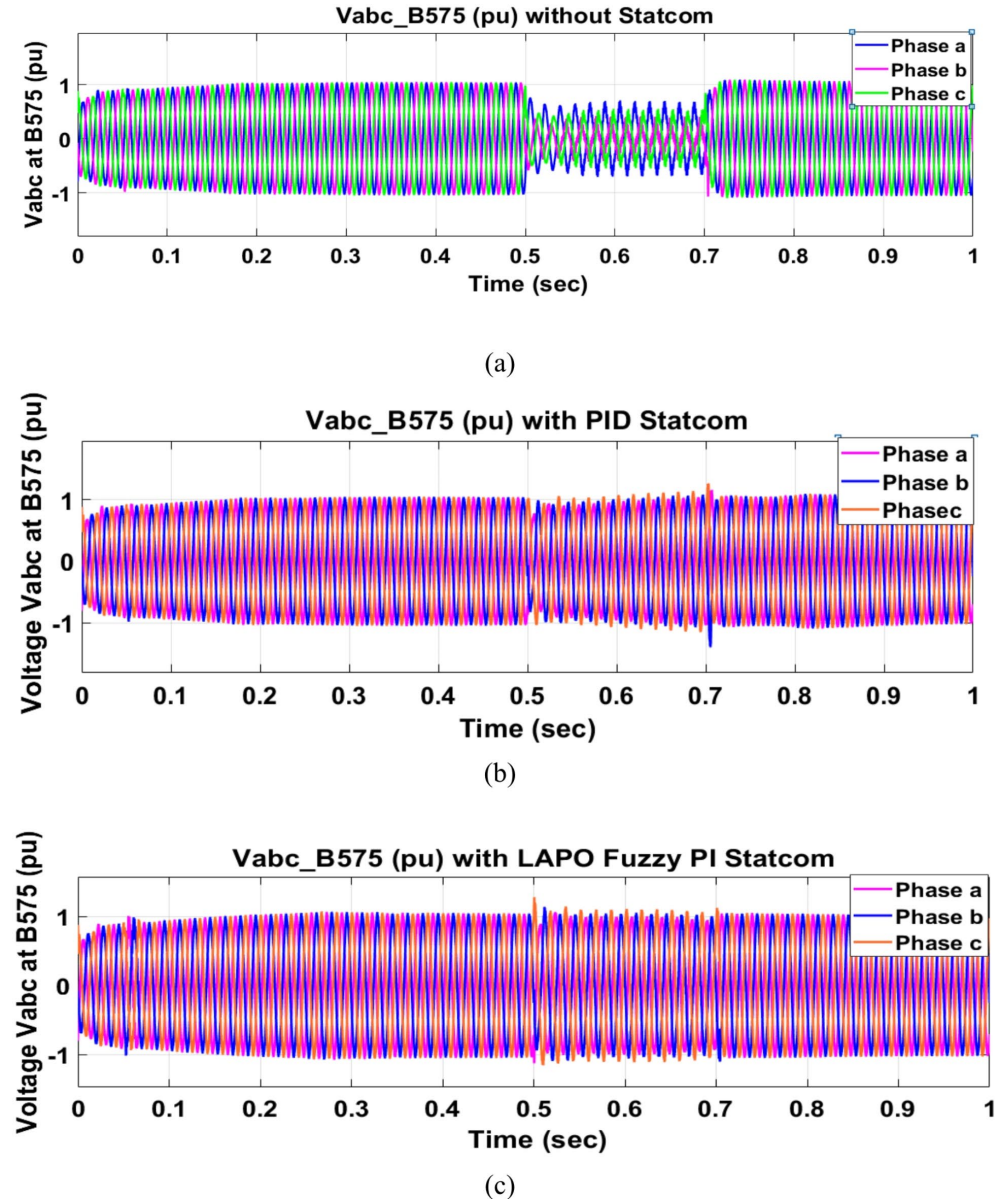
Voltage at Bus 575 through DLG. (**a**) Voltage at Bus 575 through DLG without connects STATCOM. (**b**) Voltage at Bus 575 through DLG with PID controller STATCOM. (**c**) Voltage at Bus 575 through DLG with PI Fuzzy control STATCOM.

**Fig. 34:**
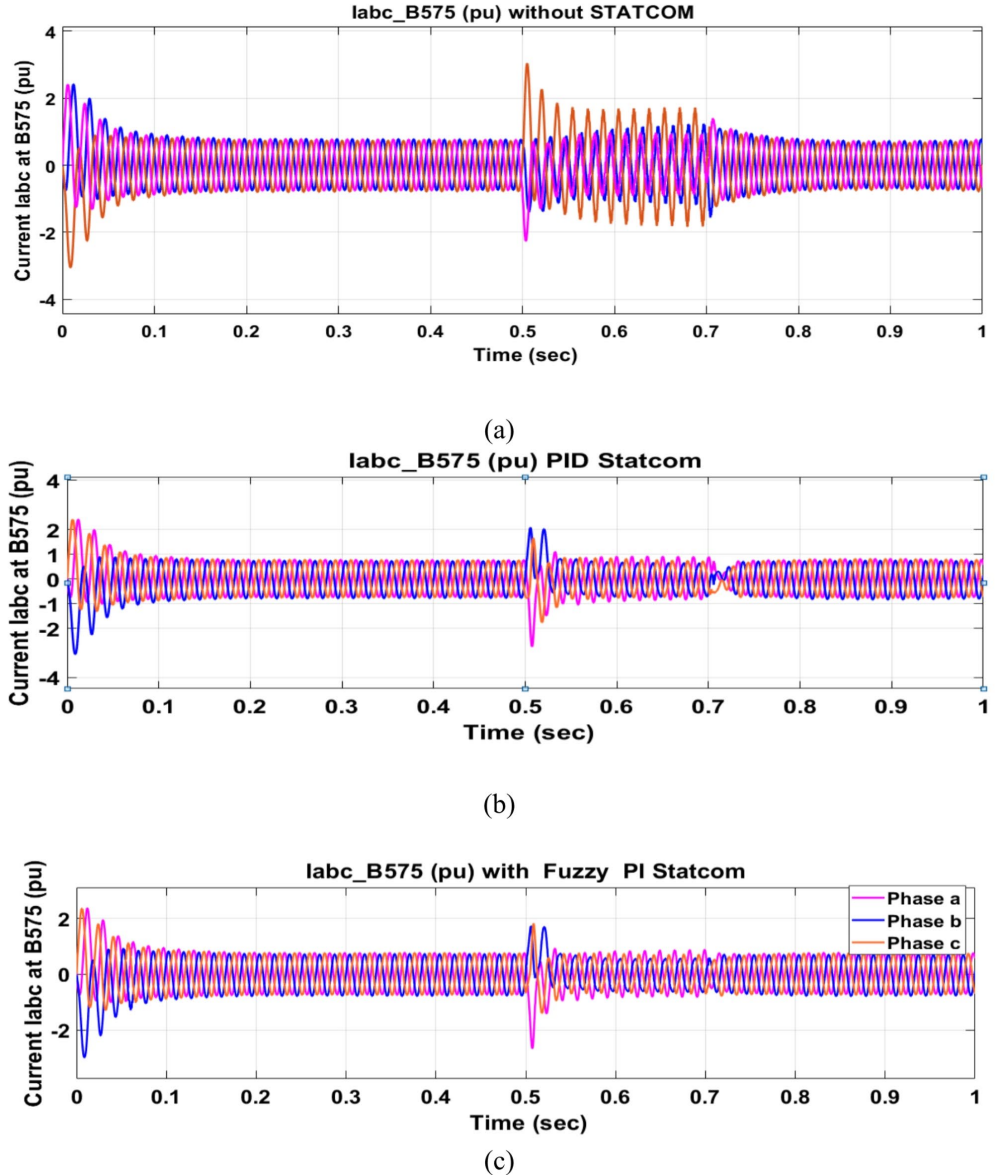
Current at Bus 575 through DLG. (**a**) Current at Bus 575 through DLG without connects STATCOM. (**b**) Voltage at Bus 575 through DLG with PID controller STATCOM. (**c**) Voltage at Bus 575 through DLG PI fuzzy control STATCOM.

**Fig. 35:**
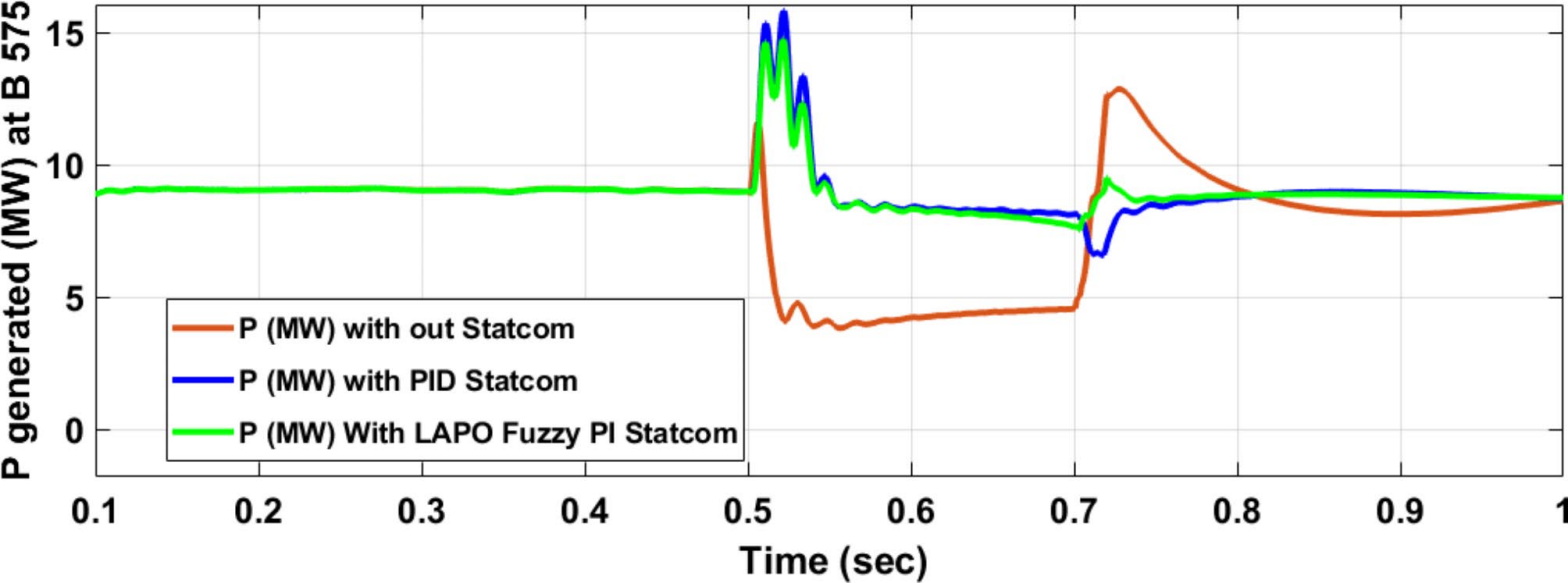
Active power generated at Bus 575 through DLG without connect STATCOM, with PID controller STATCOM and with PI Fuzzy control STATCOM.

**Fig. 36:**
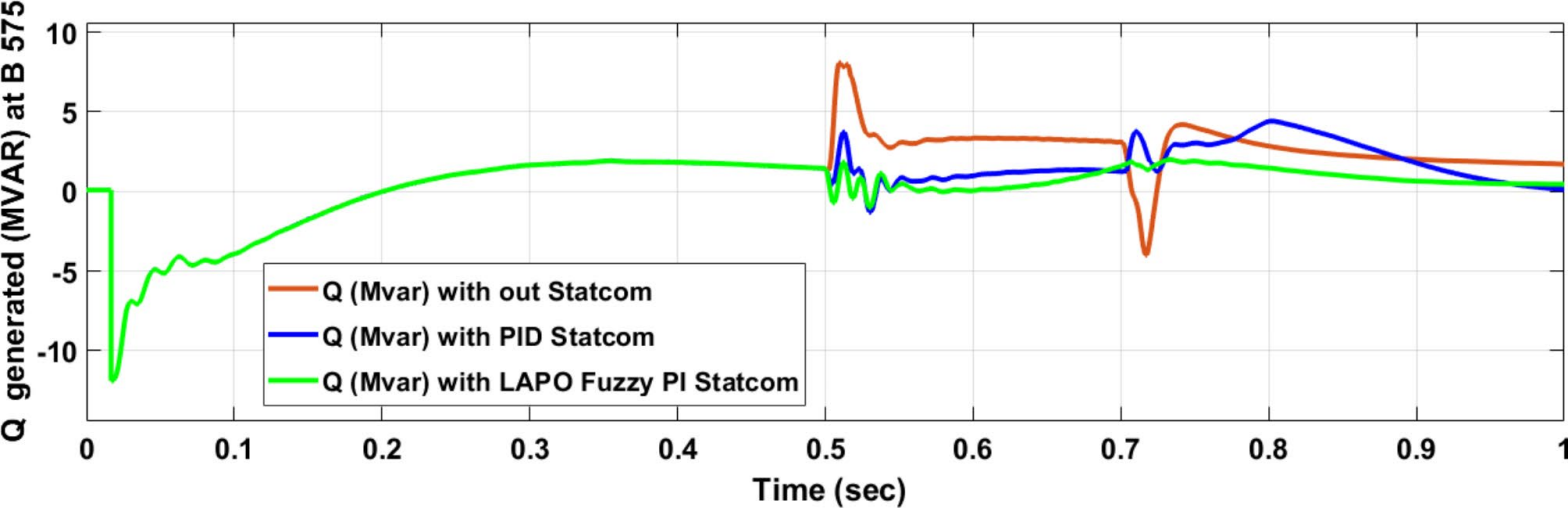
Reactive power generated at Bus 575 through DLG without connect STATCOM, with PID controller STATCOM and with PI Fuzzy control STATCOM.

**Fig. 37:**
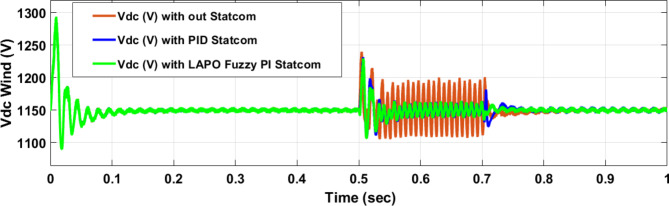
Voltage of DC link at Bus 575 through DLG without connect STATCOM, with PID controller STATCOM and with PI Fuzzy control STATCOM.

**Fig. 38:**
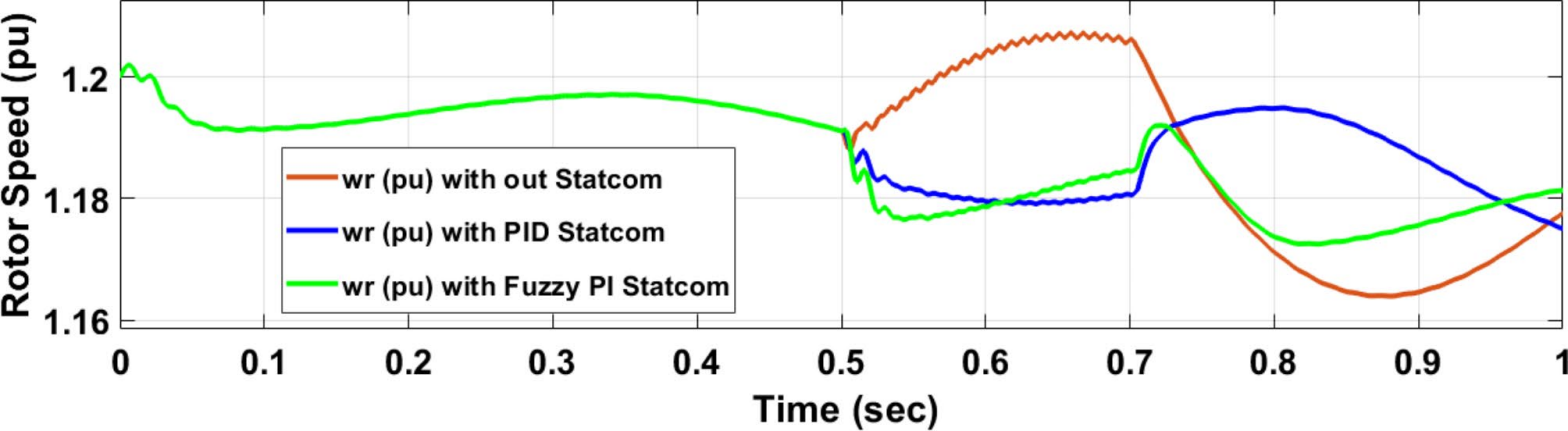
Rotor speed of DFIG through DLG without connect STATCOM, with PID controller STATCOM, and with PI Fuzzy control STATCOM.

Voltage sag occurs at bus 25 KV the period of fault between 0.5 and 0.7 s as shown in Fig. 31a when it is not connected to STATCOM and when PID and FPI STATCOM are connected voltage sag was mitigated as shown in Fig. 31b, c. Also Fig. 32a, b, c shows the current at Bus 25 KV before and after connected STATCOM to mitigate the current swell but STATCOM does not mitigate the current swell at Bus 25 KV. Voltage sag occurs at Bus 575 V when STATCOM is not connected as shown in Fig. 33a and is mitigated when STATCOM is connected as shown in Fig. 33b, c. Figure 34a–c shows the current before and after connected STATCOM. Figure 35 shows active power before and after connected STATCOM with PID, and FPI. Figure 36 shows reactive power before and after connected STATCOM with PID, and FPI. Figure 37 shows the DC link voltage before and after linked STATCOM with PID, and FPI. Figure 38 shows rotor speed of DFIG before and after linked STATCOM with PID, and FPI. Also, comparisons between the PID controller and PI Fuzzy logic controller are studied to improve LVRT as shown in Table 12. Also, Time domain analysis of DC link voltage for wind bus is shown in Table 13.

**Table 12:** Comparisons of results through DLGF at B 575 V.

Comparison cases	Active power (MW) minimum–maximum	Reactive power (MW) minimum–maximum
Without STATCOM	4.3–12.48	− 3.8–7.9
With PID STATCOM	6.68–15.6	− 1.03–3.5
With PI Fuzzy STATCOM	7.79–14.4	− 0.5–1.5

**Table 13:** The time domain analysis for DC–link voltage through DLGF at wind bus.

At t = 0.5 s				At t = 0.7 s		
Comparison cases	Settling time	Under shoot	Over shoot	Settling time	Under shoot	Over shoot
Without STATCOM	0.7255	1106.38	1236.84	0.7255	1113.27	1197.3
With PID STATCOM	0.586	1131.27	1221.39	0.7349	1130.37	1181.88
With PI Fuzzy STATCOM	0.588	1112.34	1223.12	0.7589	1138.98	1163.01

#### PV model results

**Fig. 39:**
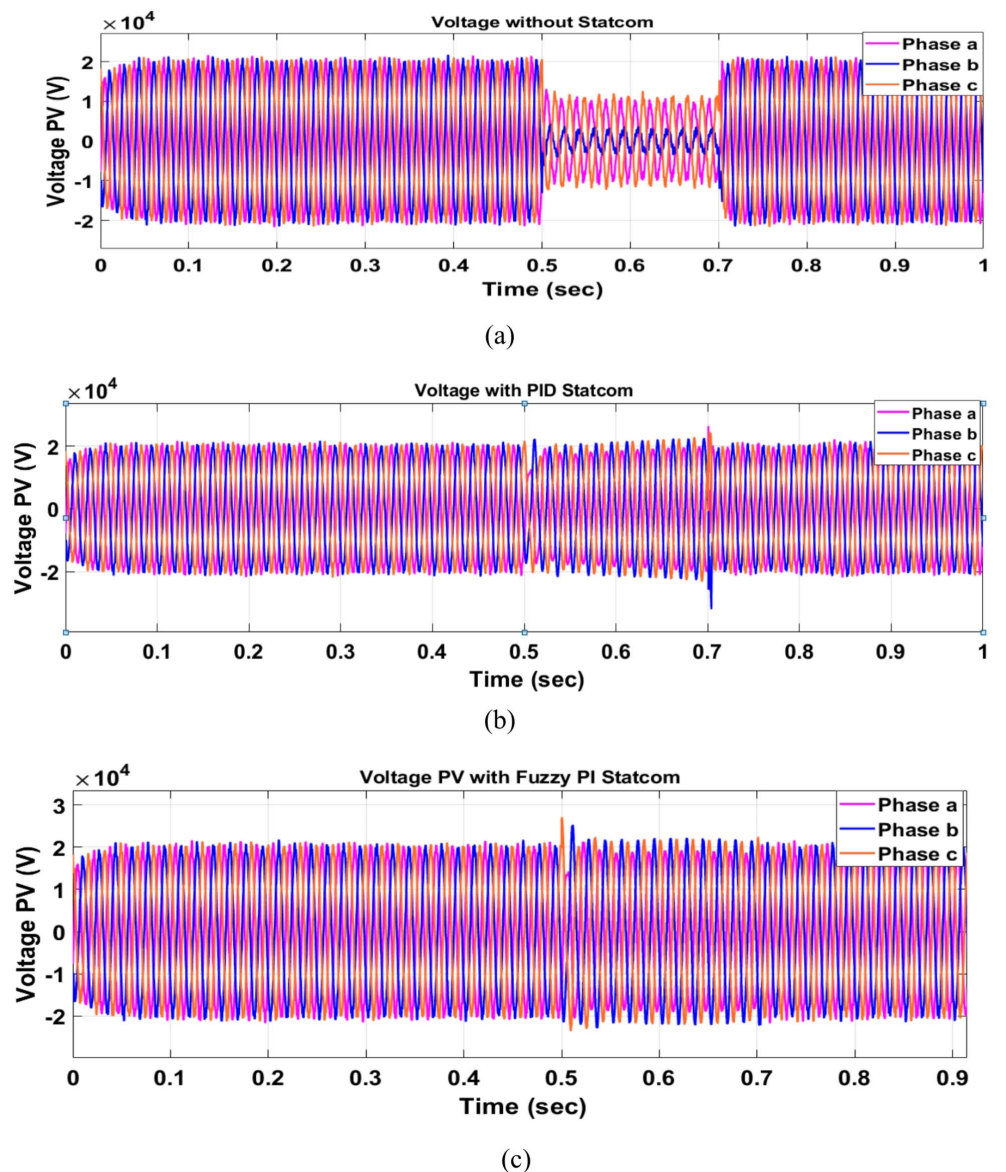
Voltage at PV Bus through DLG. (**a**) Voltage at PV Bus through DLG without connects STATCOM. (**b**) Voltage PV Bus through DLG with PID controller STATCOM. (**c**) Voltage PV Bus through DLG with PI Fuzzy control STATCOM.

**Fig. 40:**
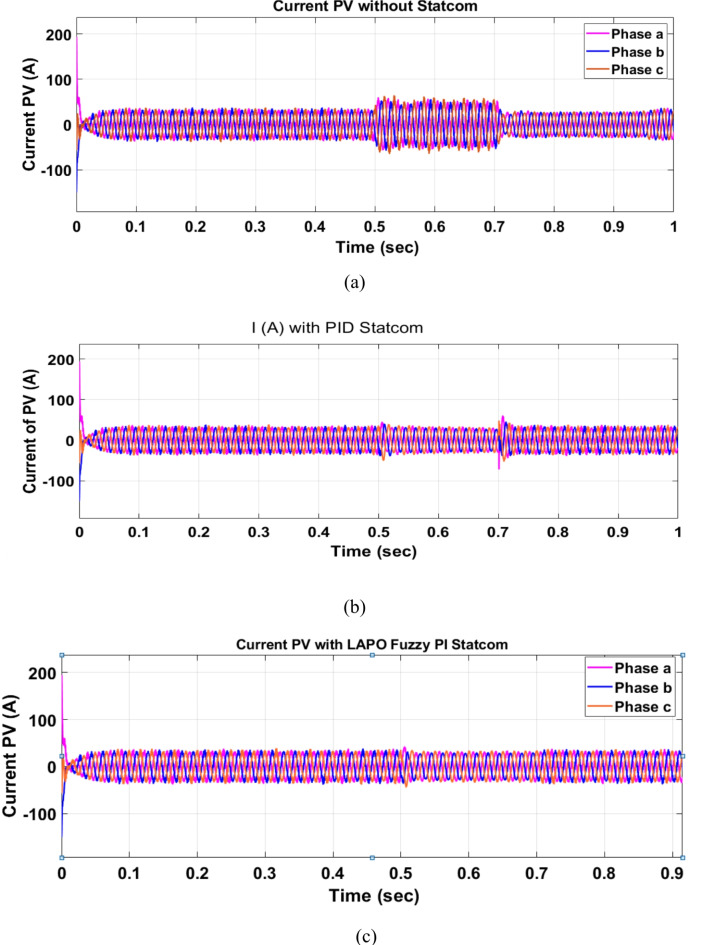
Current PV at Bus 25 through DLG. (**a**) Current PV at Bus 25 through DLG without connects STATCOM. (**b**) Current PV at Bus 25 through DLG with PID controller STATCOM. (**c**) Current PV at Bus 25 through DLG with PI Fuzzy control STATCOM.

**Fig. 41:**
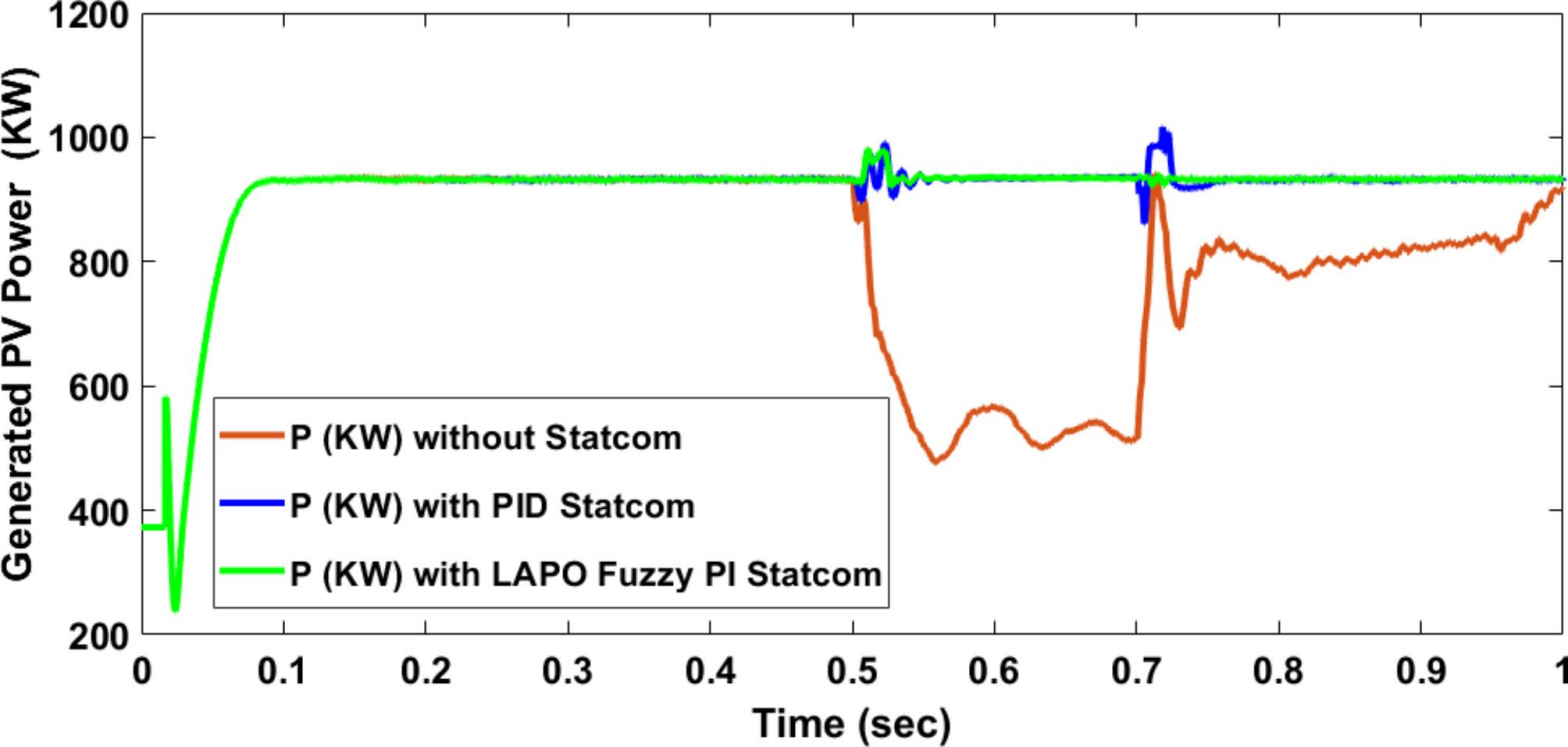
Generated PV active power through DLG without connect STATCOM, with PID controller STATCOM and with PI Fuzzy control STATCOM.

**Fig. 42:**
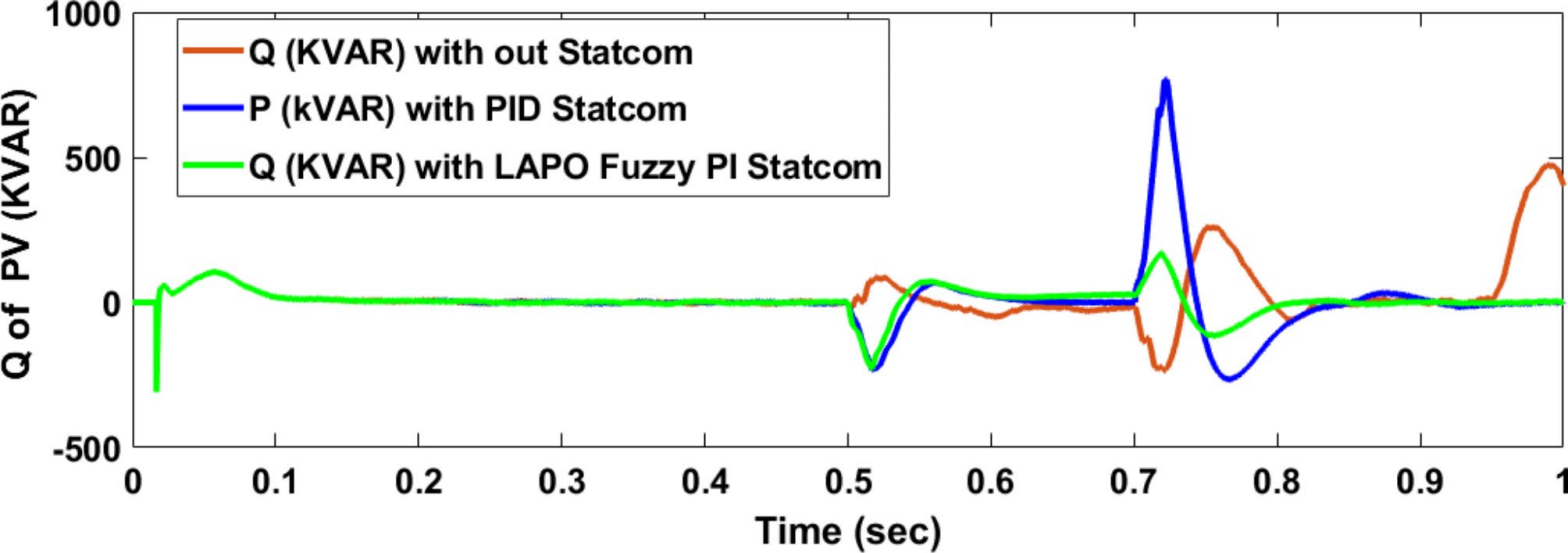
Generated PV reactive power through DLG without connect STATCOM with PID controller STATCOM and with PI Fuzzy control STATCOM.

**Fig. 43:**
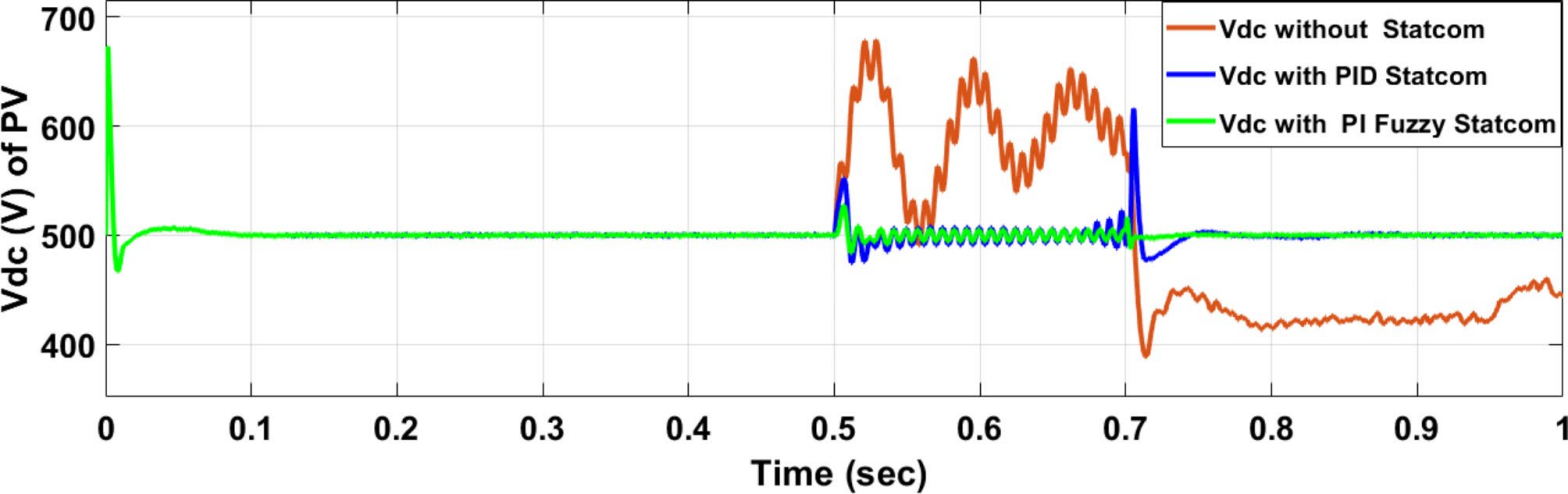
Voltage PV of DC link through DLG without connect STATCOM, with PID controller STATCOM, and with PI Fuzzy control STATCOM.

Voltage sag occurs at the PV bus at the period of fault as shown in Fig. 39a and when PID and FPI STATCOM connected voltage sag was mitigated as shown in Fig. 39b, c. Also, Fig. 40a–c shows the current before and after connected STATCOM to mitigate voltage swell. Figure 41 shows active power before and after connected STATCOM with PID, and FPI. Figure 42 shows Reactive power before and after connected STATCOM with PID, and FPI. Figure 43 shows the DC Link voltage before and after linking STATCOM with PID, and FPI. Also, comparisons between the PID controller and PI Fuzzy logic controller are studied to improve LVRT as shown in Table 14. Also, time domain analysis of DC link voltage for PV bus is shown in Table 15.

**Table 14:** Comparisons of results through DLGF at PV bus.

Comparison cases	Active power (KW) minimum–maximum	Reactive power (KVAR) minimum–maximum
Without STATCOM	506–908	− 222.5–258
With PID STATCOM	865–1016	− 208–760
With PI Fuzzy STATCOM	934–980	− 194–150

**Table 15:** The time domain analysis for DC–link voltage through DLGF at PV bus.

At t = 0.5 s				At t = 0.7 s		
Comparison cases	Settling time	Under shoot	Over shoot	Settling time	Under shoot	Over shoot
Without STATCOM	0.7599	518.91	677.421	0.7796	388.320	606.72
With PID STATCOM	0.52955	476.14	541.628	0.7462	478.8	610.74
With PI Fuzzy STATCOM	0.53405	486.863	520.233	0.7101	490.69	512.13

#### PV/Wind hybrid system results

**Fig. 44:**
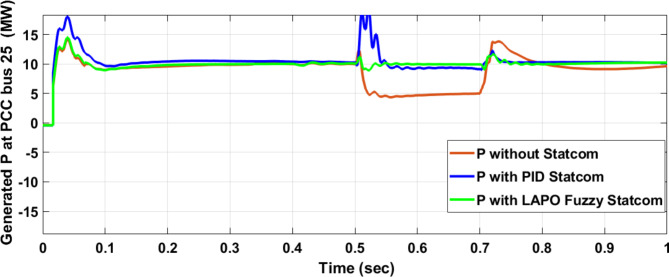
Active power generated at Bus PCC through DLG without connecting STATCOM with PID controller STATCOM and with PI Fuzzy control STATCOM.

**Fig. 45:**
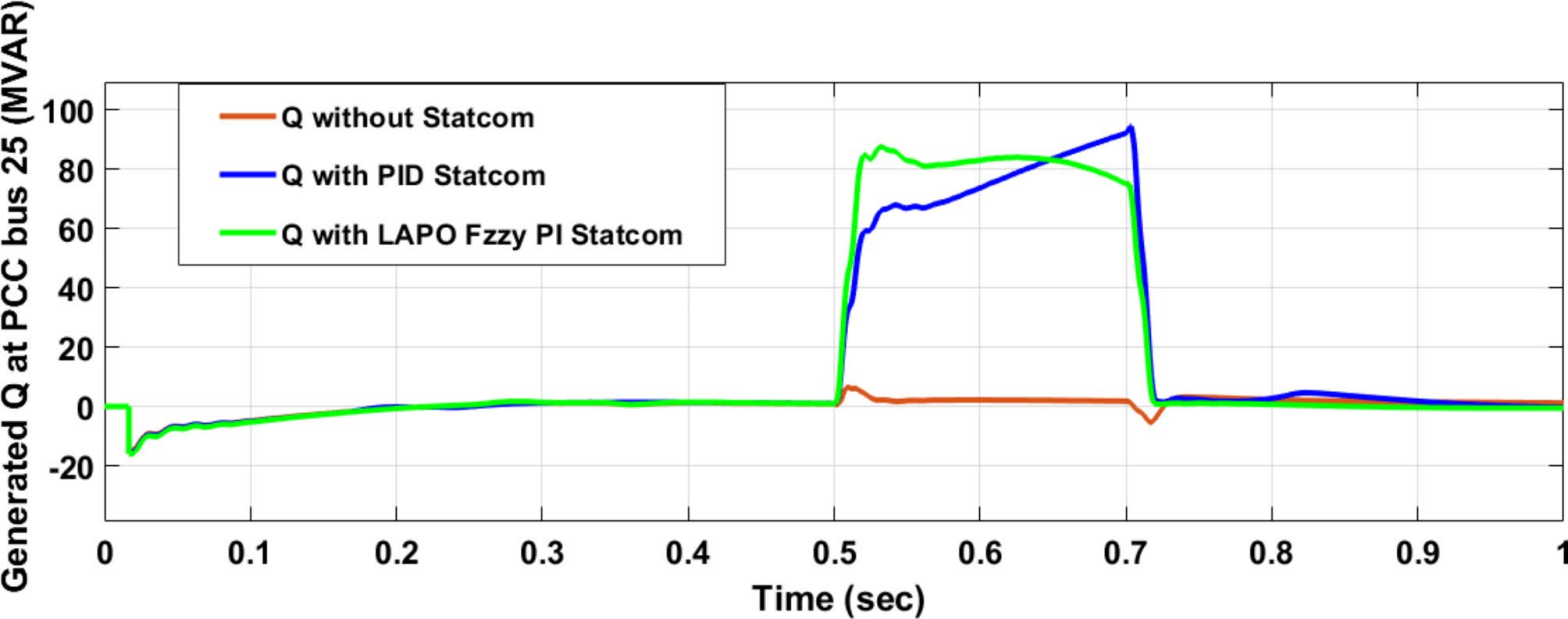
Reactive power generated at Bus PCC through DLG without connecting STATCOM with PID controller STATCOM and with PI Fuzzy control STATCOM.

**Fig. 46:**
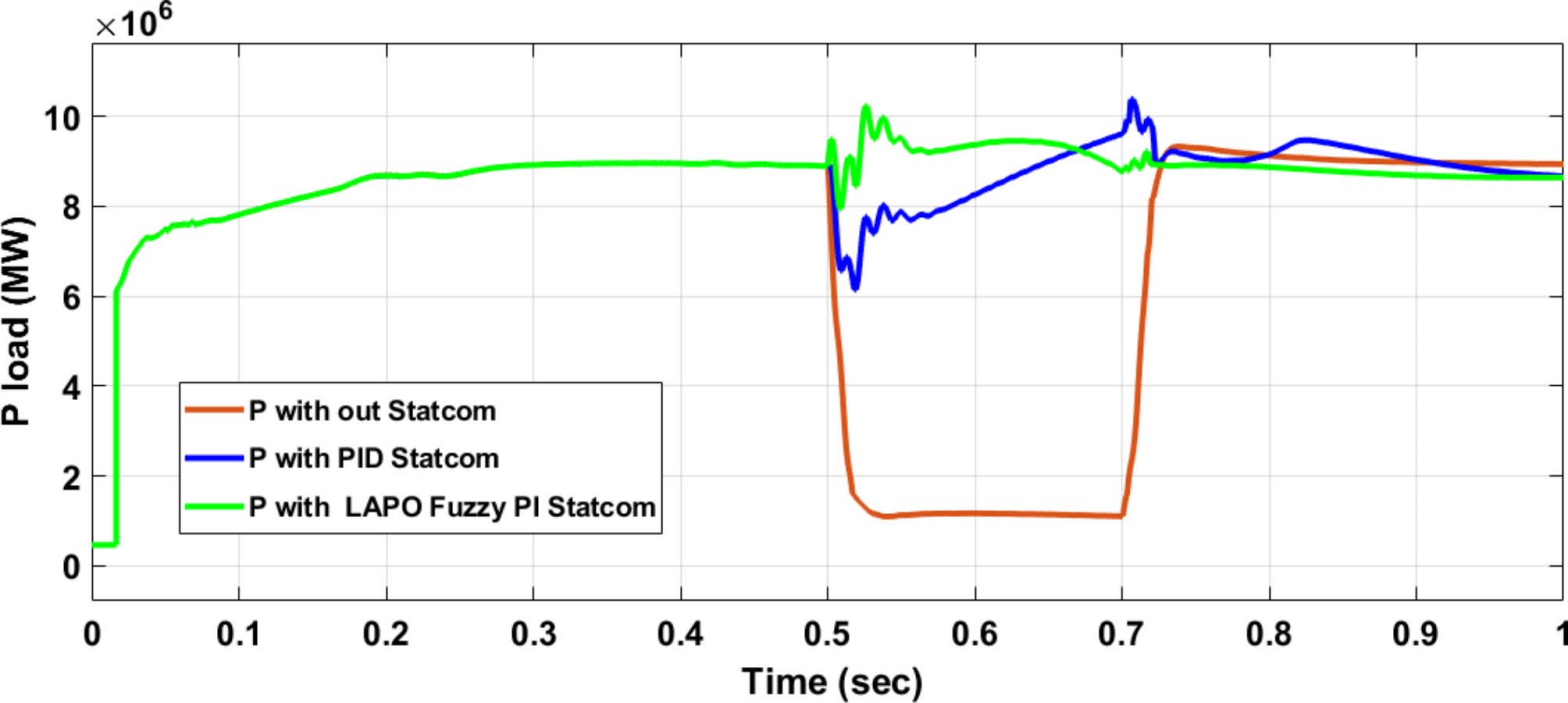
Active power of load through DLG without connect STATCOM, with PID controller STATCOM, and with PI Fuzzy control STATCOM.

**Fig. 47:**
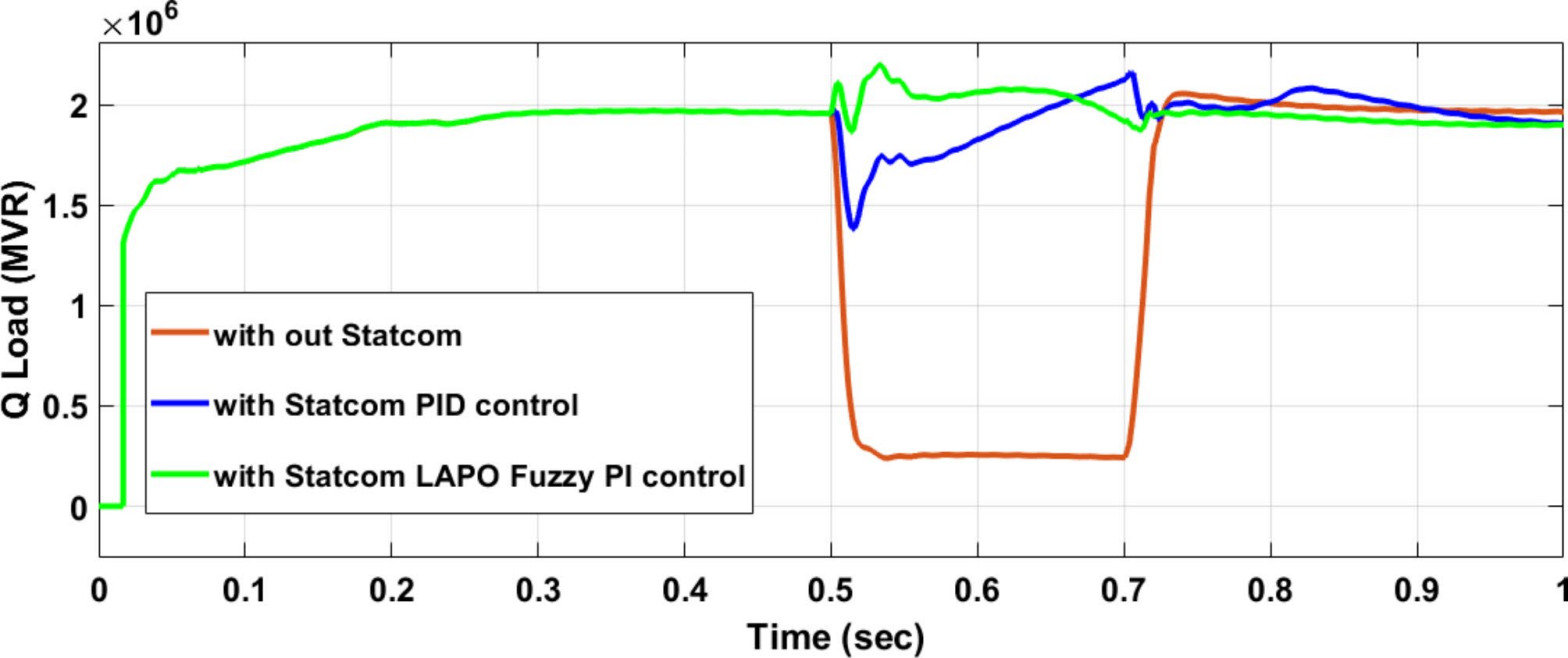
Reactive power of load through DLG without connect STATCOM, with PID controller STATCOM and with PI Fuzzy control STATCOM.

**Fig. 48:**
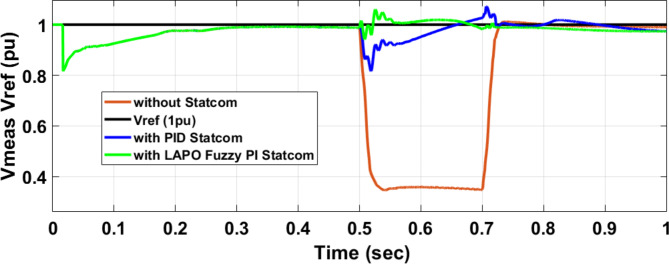
V_meas,_ V_ref_ STATCOM through DLG without connect STATCOM, V_ref_, with PID controller STATCOM and with PI Fuzzy control STATCOM.

**Fig. 49:**
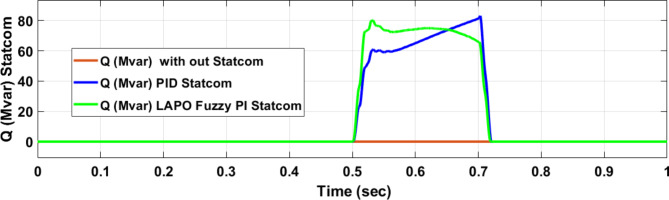
Reactive power generated at STATCOM through DLG without connect STATCOM, with PID controller STATCOM, and with PI Fuzzy control STATCOM.

Figure 44 shows active power of hybrid PV/wind generated at Bus 25 before and after connected STATCOM in case PID, and FPI. Figure 45 shows reactive power before and after connecting STATCOM with PID, and FPI. Figure 46 shows the active power of the load before and after connecting STATCOM. Figure 47 shows the reactive power of load before and after connected STATCOM with PID, and FPI. Figure 48 shows V_meas_, V_ref_ where V_meas_ is voltage measured and V_ref_ is voltage reference at bus STATCOM before and after STATCOM connected with PID, and FPI. Also, Fig. 49 shows reactive power generated at Bus STATCOM with PID, and FPI. Comparisons between the PID controller and PI Fuzzy logic controller are studied to improve LVRT as shown in Table 16. Also, comparisons between the PID controller and PI Fuzzy logic controller are studied at STATCOM Bus to improve LVRT as shown in Table 17.

**Table 16:** Comparisons of results through DLGF at hybrid PV/wind bus 25 KV.

Comparison cases	Active power (MW) minimum–maximum	Reactive power (MVAR) minimum–maximum	*P* Load (MW)	Q (MVAR)
Without STATCOM	4.5–13.5	59.55–94.56	1.16	0.25
With PID STATCOM	8.8–17.7	59.55–94.56	8.2	1.7
With PI Fuzzy STATCOM	9.1–11.2	75–87.5	9.1	2.05

**Table 17:** Comparisons of results through DLGF at STATCOM Bus.

Comparison cases	Vmeas	Vref	Reactive power
Without STATCOM	0.35	1	0
With PID STATCOM	0.83–1.07	1	60–81
With PI Fuzzy STATCOM	0.977–1.05	1	70.6–80.2

## Conclusion

This paper discusses the problem of LVRT and its effect on the electrical grid connected to the PV/wind hybrid system. Applying 100 MVAR STATCOM linked to a hybrid system that consists of a 1 MW PV station and 9 MW wind farm to improve LVRT, voltage stability, and power quality. The LAPO optimization method is used to adjust the control values of PI FLC. The simulation and results applied to STATCOM reduce voltage sagging, compensate active power and reactive power of wind and PV station, and protect the DC-link voltage of PV and wind from over-voltage and oscillation. In the case of a three-phase fault, STATCOM with PI FLC compensates for voltage by 98.5% compared with PID which compensates for voltage by 93.6%. STATCOM with PI FLC gives 98.8 MVAR reactive power compared with STATCOM with PID gives 86 MVAR reactive power. In the second case, double line to ground fault STATCOM with PI FLC compensates voltage by 97.7% compared with PID which compensates voltage by 83%. Also, STATCOM with PI FLC gives 75 MVAR reactive power compared with STATCOM with PID gives 63 MVAR reactive power. The time domain parameters like settling time, undershoot, and overshoot for DC-link voltage at the wind bus and PV bus are calculated. The comparison between the PID STATCOM controller method, and the PI Fuzzy STATCOM controller method confirms the effective performance of the STATCOM controller but PI FLC gives better performance in improving LVRT capability, voltage stability, and power quality.

## Data Availability

Data is provided within the manuscript or supplementary information files.

## List of symbols

LVRT: Low Voltage Ride Through
DFIG: Doubly Fed Induction Generator
STATCOM: Static Compensator
PCC: Point Common Coupling
AIC: Artificial Intelligence Control
PI FLC: Proportional–Integral Fuzzy Logic Control
LAPO: Lightning Attachment Procedure Optimization Algorithm
SEIG: Self-Excited Induction Generator
DVR: Dynamic Voltage Restorer
ANN: Artificial Neural Network
WTGS: Wind Turbine Generator System
GA: Genetic Algorithm
RSC: Rotor side converter
GSC: Grid side converter
${I}_{pv}, {V}_{ph}$: Current, Voltage of PV panel
${R}_{s }, {R}_{sh}$: Series, Shunt resistances
$a,\text{K}$: Ideality factor, Boltzmann constant (1.38 × 10^−23^ J)
n, T: Number of cell in series, Temperature of PV cell
Q: Electron charge (1.602 × 10^−19^ C)
${I}_{ph}, {I}_{o},{I}_{d}$: Photocurrent and reverse saturation current, and diode current
${V}_{d-inv}, {V}_{q-inv}$: Inverter voltage component of d–q axis
${V}_{d}, {V}_{q}$: Grid voltage components of d–q axis
${I}_{d}, {I}_{q}$: Inverter current component of d–q axis
${L}_{f}, {R}_{f}$: Resistance and inductance for filter of PV
$\left(\lambda ,\beta \right)$: Tip speed ratio of rotor blade and blade pitch angle
$\Delta t{,C}_{2}$: Duration of grid voltage sag and PV DC-link capacitor
$Vdc2,Vdc2 - {\text{f}}$: DC-link voltage before and during voltage sag of grid
${V}_{f},{I}_{g}$: RMS voltage of grid through sagging of voltage, Inject current from PV farm
${P}_{m},\text{Pe}$: Mechanical and electrical power of wind
${\omega }_{r},{\omega }_{e}$: Mechanical angular speed of rotor and electrical angular speed of stator
$${H}_{g}$$: Inertia constant
${C}_{p},{v}_{w}, \rho , {A}_{t}$: Wind performance coefficient, velocity of wind, density of air, and area swept out by blades of wind turbine
${L}_{choke}$: Filter inductance of DFIG turbine
${i}_{dg}, {i}_{qg}$: GSC current component of d–q axis
${i}_{dr}, {i}_{qr}$: RSC current component of d–q axis
${L}_{r}, {L}_{m}$: Inductance of rotor, mutual inductance
${i}_{ms}$: Current of stator magnetizing
${v}_{g}{,v}_{st}$: Voltage grid and voltage of STATCOM
$\alpha$: Phase angle between grid voltage and STATCOM voltage
${x}_{tr}$: Coupling transformer Leakage reactance between electrical grid and VSI
${P}_{g},{Q}_{g}$: Active and reactive power of electrical grid
${V}_{meas},{V}_{ref}$: Measured voltage and reference voltage
